# Sustainable Biomass‐Derived Single‐Atom Catalysts for Fenton‐Like Catalysis

**DOI:** 10.1002/smll.202504746

**Published:** 2025-07-09

**Authors:** Selusiwe Ncube, Zhihao Tian, Jingkai Lin, Huayang Zhang, Shaobin Wang, Wenjie Tian

**Affiliations:** ^1^ School of Chemical Engineering The University of Adelaide North Terrace Adelaide SA 5005 Australia

**Keywords:** biomass, Fenton‐like catalysis, single atom catalyst, transition metals, water remediation

## Abstract

Single‐atom catalysis has generated significant interest in catalysis, particularly for environmental remediation. Pursuing desired single atom catalysts (SACs) for practical application hinges on the simplicity of synthesis methods, the choice of precursor materials, cost‐effectiveness, and catalytic performance. Consequently, natural resources like biomass have been harnessed to prepare SACs, driving continuous efforts to establish controlled and scalable synthesis techniques. This comprehensive review encompasses a broad exploration of diverse synthesis methods employed for crafting SACs derived from biomass, intended for environmental catalysis by Fenton‐like reactions, employing peroxymonosulphate (PMS), peroxydisulphate (PDS), or hydrogen peroxide (H_2_O_2_) as the oxidants. It highlights how these innovations can stimulate advancements in catalyst design, shaping the landscape of sustainable and efficient micropollutant remediation. In addition, the review summarizes the links between single‐atom sites on biomass‐derived SACs and catalytic mechanisms, imparting insights into the origins of catalytic activity. Finally, this review proposes the prevailing challenges and prospects in this field. It is anticipated that this review would inform the development of biomass‐derived SACs and their application in environmental remediation by guiding the selection of synthesis methods and addressing the key areas for improvement highlighted in future research.

## Graphical abstract

The review comprehensively examines synthesis methods for biomass‐derived transition metal‐based singleatom catalysts (SACs). It narrows focus to Fenton‐like reactions, emphasizing how synthesis‐property relationships and active site configurations influence reaction mechanisms and catalytic performance, offering insight into the design of SACs for water treatment applications.

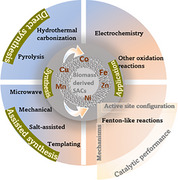

## 1 Introduction

Rapid industrialization and human activities have culminated in detrimental waste disposal practices, resulting in severe environmental pollution.^[^ ^1^ ^]^ The ongoing depletion of fossil fuels exacerbates these environmental challenges and significantly impedes the development of a sustainable society.^[^ ^2^ ^]^ Among several strategies for developing a cleaner environment, catalysis stands out as a pivotal tool in facilitating the transition toward sustainability.^[^ ^3^ ^]^ Extensive research efforts have been directed toward heterogeneous catalysis, in contrast to homogenous catalysis, primarily due to their inherent advantages, such as enhanced stability and recyclability.^[^ ^4^ ^]^ This highlights the importance of developing catalysts that not only drive essential catalytic reactions but also contribute to the broader goal of achieving a cleaner, more sustainable future. Single atom catalysts (SACs) are promising materials due to their high catalytic activity and atom utilization, offering advantages in both heterogeneous and homogeneous catalysis.^[^ ^5^ ^]^ To prevent metal atom aggregation and maintain dispersion, various carrier materials have been employed, including metal‐organic frameworks (MOFs),^[^ ^6^, ^7^ ^]^ metal‐based supports,^[^ ^8^, ^9^ ^]^ and carbon‐based materials.^[^ ^10^, ^11^, ^12^, ^13^, ^14^ ^]^ While these supports have demonstrated effectiveness in anchoring single atoms, each comes with limitations that hinder their practical application. MOFs are often unstable at elevated reaction conditions.^[^ ^15^ ^]^ Metal‐based supports, though potentially promising, remain underexplored, resulting in limited performance and stability in SAC configurations. Metal oxides, while chemically stable, generally exhibit lower single‐atom loading compared to carbon‐based materials.^[^ ^16^ ^]^ Carbon materials like graphitic carbon nitride suffer from low SSA and dense structures due to the limitations of the synthesis methods.^[^ ^17^ ^]^ Graphene oxide, on the other hand, provides a high surface area but its weak metal‐support interactions compromise atom stability.^[^ ^18^ ^]^ Furthermore, its separation and recovery from aqueous reaction systems pose additional challenges,^[^ ^18^ ^]^ limiting reusability. SACs on their own are limited by low active sites and mass loading,^[^ ^19^ ^]^ which restricts their scalability. The combined limitations of both SACs and their conventional supports have prompted interest in alternative materials that can enhance both stability and performance. Biomass is an abundant and low‐cost material,^[^ ^20^ ^]^ with the potential to serve as a precursor for supports in the synthesis of SACs.^[^ ^21^ ^]^ Thus, biomass‐derived supports have emerged as a compelling solution, offering not only sustainability but also structural tunability, surface functionality, and improved atom anchoring ability. The natural heteroatom content and porous carbon frameworks in some biomass sources make them promising materials for supports and addressing the current limitations of SACs while also contributing to the development of green and cost‐effective catalytic systems. Biomass‐derived SACs have emerged in various catalytic applications like redox reactions in electrochemistry,^[^ ^22^, ^23^ ^]^ Fenton‐like reactions,^[^ ^24^, ^25^ ^]^ and other oxidation processes.^[^ ^26^, ^27^ ^]^ However, the tendency of metal atoms to aggregate necessitates precise control over synthesis strategies.^[^ ^28^ ^]^ With different biomass species, various synthesis routes contribute uniquely to the fundamental properties of SACs, e.g., morphology, crystallinity, and pore structure.^[^ ^29^, ^30^, ^31^ ^]^ These features are essential for tailoring SACs to specific application areas, and recent reviews have explored their synthesis and catalytic performance in different sectors. Some studies have examined SACs broadly,^[^ ^32^ ^]^ discussing strategies to engineer the microenvironments of metal active sites to enhance catalytic performance.^[^ ^33^ ^]^ Others have compared SACs with ultra‐small atom clusters and nanoparticles in Fenton‐like catalysis.^[^ ^34^ ^]^ One study focused on carbon‐based SACs in Fenton‐like catalysis, highlighting the relationship between material structure and the generation of reactive oxygen species (ROS).^[^ ^35^ ^]^ Reviews specifically on biomass‐derived SACs have addressed various aspects, such as synthesis strategies, characterization techniques and applications,^[^ ^36^ ^]^ synthesis methods and catalytic mechanisms relevant to electrocatalysis,^[^ ^37^ ^]^ roles in oxygen and hydrogen evolution reactions,^[^ ^38^ ^]^ and the influence of biomass precursors on their morphology and structure.^[^ ^39^ ^]^ Despite these advancements, gaps remain in achieving a comprehensive understanding of biomass‐derived SACs. There remains a need to understand the fundamental synthesis‐property‐structure relationships, underlying catalytic mechanisms and how specific coordination environments influence catalytic performance in Fenton‐like catalysis, providing insights for the design of biomass‐derived SACs.

As such, the review aims to address these gaps comprehensively while also highlighting recent advancements in the field. This review comprehensively integrates synthesis methods, characterization techniques, and catalytic applications of biomass‐derived SACs in Fenton‐like reactions for the efficient degradation of organic micropollutants. The review emphasises synthesis‐property‐structure relationships and analyses how the metal coordination environments in biomass‐derived SACs can be engineered to enhance performance in Fenton‐like reactions. By drawing connections between biomass precursor selection, synthesis‐property‐structure relationships, metal coordination structure and resulting catalytic mechanisms, the review provides a comprehensive framework that addresses gaps in previous literature. Finally, the review summarizes the current research, outlines key challenges, and gives future perspectives to guide continued research in this area.

## 2 Synthesis Strategies for Biomass‐Derived SACs

**Scheme 1:**
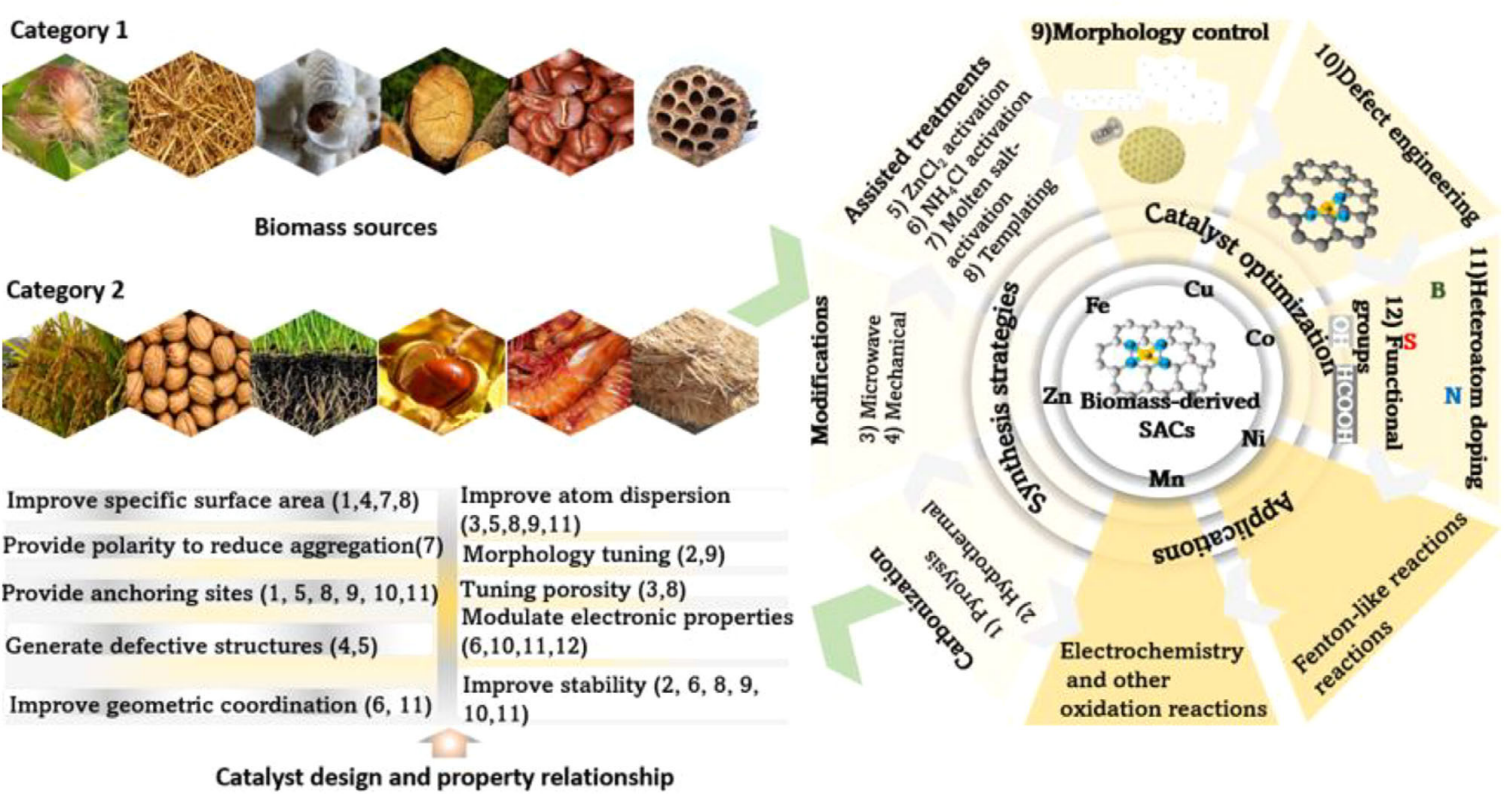
An overview of the different synthesis methods, catalyst properties, and applications of SACs from different biomass precursors used in this review.

This section provides an overview of the various synthesis approaches employed in the fabrication of biomass‐derived SACs. Biomass precursors of diverse sources, such as plants,^[^ ^40^, ^41^ ^]^ animal waste,^[^ ^27^, ^42^ ^]^ and algae,^[^ ^43^, ^44^ ^]^ can be used directly in the fabrication of SACs (**Scheme** **1** (category 1)). The synthesis strategies are highly dependent on the composition of the precursors.^[^ ^45^ ^]^ Alternatively, biomass can be converted into a carbon support first with enhanced structural properties, followed by effective anchoring of single atoms (Scheme 1 (category 2)). Generally, the treatment methods of biomass sources and metal precursors encompass hydrothermal treatment,^[^ ^46^, ^47^ ^]^ template‐assisted treatment,^[^ ^41^, ^48^ ^]^ pyrolysis,^[^ ^49^, ^50^ ^]^ mechanical‐assisted treatment,^[^ ^51^, ^52^ ^]^ etc., offering unique pathways for tuning the structure of SACs (Scheme 1).

### 2.1 Direct Conversion of Biomass to SACs

Various synthesis strategies are employed to transform biomass into SACs. The synthesis strategy guides further research on the possibility of combining the functionalization, templating, activation, and graphitization agents to form catalysts with ultrahigh surface areas. Raw biomass precursors can be converted into SACs through two main pathways: direct pyrolysis, which relies on the inherent composition and properties of the raw biomass precursors, or pyrolysis combined with external metal or dopants to enhance catalytic performance. For the latter, the mixing methods vary, which can include solution‐based wet mixing by adsorbing external agents on biomass, solid‐state mechanical mixing, or even introducing metals or dopants through irrigation during the plant growth process. Some chelating, activation reagents like Zn salts, NH_4_Cl, molten salts, hard‐templates, soft templates, and microwave treatment can also be introduced to improve the mixing with metal precursors or to enhance the physiochemical properties of carbon matrix.

#### 2.1.1 Pyrolysis of Biomass for SAC Synthesis With or Without External Metal/Dopants

Direct pyrolysis is a simple and scalable method to convert biomass precursors rich in nitrogen, carbon, and metal species into SACs. Thermal decomposition facilitates metal‐nitrogen coordination within carbon matrices, leading to atomically dispersed metals. The effectiveness of this approach depends significantly on the biomass composition, coordination environment, and thermal treatment conditions. The direct pyrolysis of biomass, as well as pyrolysis involving additional dopants or metal salts, have all been reported.

Biomass with inherently low nitrogen content, such as *Enteromorpha*, yields limited metal loading and suboptimal coordination environments. Peng et al.^[^ ^53^ ^]^ pyrolyzed pre‐dried *Enteromorpha*, which inherently contained Fe and N at 900 °C, followed by leaching treatment to synthesize Fe clusters and single‐atom Fe on N‐doped carbon. The elevated temperature favored the formation of more pyridinic N, thus leading to more active Fe–N_2_–O_2_ sites for catalytic Fenton‐like reaction. However, owing to the low N content in *Enteromorpha* and the relatively weak coordination with Fe, only 0.84 wt.% metal loading was realized. Li et al.^[^ ^54^ ^]^ used residues of a heavy metal hyperaccumulator plant, Sedum alfredii, sampled from a Zn‐contaminated site and enriched with Fe to synthesize Fe─N─C SACs. The plant material was pyrolyzed at 800 °C for 2 h, facilitating Zn evaporation, leading to the self‐dispersion of Fe atoms and the formation of Fe─N─C SACs with FeN_3_O_1_ sites, responsible for the catalytic activity in Fenton‐like reactions. The catalyst had an optimal Fe loading of 1.41 wt.%, SSA of 25.52 m^2^g^‒1^, and a smooth‐edged sheet structure with wrinkled, graphene‐like layers.

For biomass with sufficient heteroatom content but limited or no metal content, external metal reagents are often introduced for SAC synthesis, usually by dispersing ground biomass with aqueous solution containing metal salts or dopants. In solution‐based processes, Cui and co‐workers employed the moderate heteroatom content (N and S groups) of spent coffee grounds by mixing them with aqueous Co^2+^ solution for 24 h before separating the Co‐adsorbed biomass by centrifugation.^[^ ^24^ ^]^ The dried mixture was then pyrolyzed at 500 °C in N_2_. The mild synthesis temperature and inherent N and S species in spent coffee grounds allowed for the dispersion and coordination with Co atoms, forming well‐distributed Co atoms existing as Co–N_3_S_1_ sites in Co–N–S–C SAC with an SSA of 293.99 m^2^g^−1^ for Fenton‐like reaction. Chitosan has abundant amino groups, enabling strong metal ion capture. Song et al. synthesized Co–N–C SACs at 600 °C by one‐pot pyrolysis process of chitosan, CoCl_2_.6H_2_O, and acetic acid.^[^ ^55^ ^]^ The resulting catalyst had a CoN_3_ configuration, and in comparison, a catalyst prepared under similar conditions but heated only to 500 °C in a 25% NH_3_/Ar atmosphere showed a CoN_4_ configuration. Thus, a higher temperature promoted the partial release of N‐containing elements, favoring the formation of unsaturated CoN_3_ sites at 600 °C. Temperatures above 600 °C led to the formation of metallic Co phases, reducing single‐atom dispersion. The pyrolysis temperature thus directly influenced the metal coordination environment, affecting the catalyst performance for Fenton‐like reactions.

**Figure 1:**
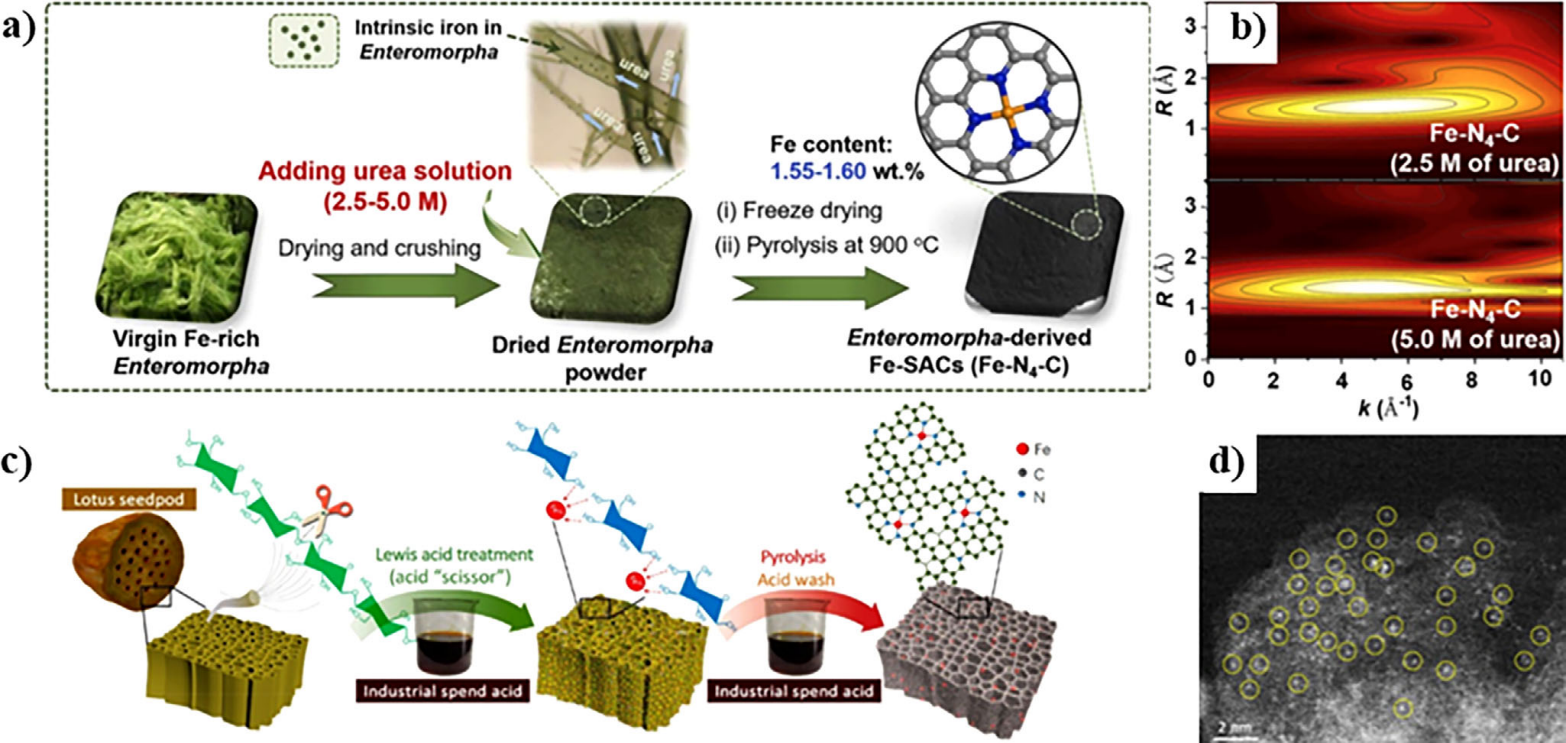
Direct pyrolysis synthesis strategy for biomass‐derived SACs. a) Schematic illustration for the synthesis process of Fe─N─C SACs from *Enteromorpha* after urea saturation and b) Wavelet transform (WT) contour plots. Reproduced with permission.^[^ ^56^ ^]^ Copyright 2023, Elsevier. c) Schematic illustration for the preparation of Fe─N─C SACs from lotus seedpod and (d) corresponding High‐angle annular dark‐field scanning transmission electron microscopy (HAADF‐STEM) image. Reproduced with permission.^[^ ^60^ ^]^ Copyright 2023, American Chemical Society.

For some biomass with sufficient metal but low heteroatom contents, external dopants can be introduced. Fe exists in many biomass plants like *Enteromorpha* in nature. However, direct pyrolysis would inevitably result in the aggregation of Fe species to form Fe nanoclusters due to the lack of coordination elements like N. Urea saturation treatment emerges as a particularly effective method for enhancing nitrogen content in biomass precursors. Yin et al. transformed urea‐saturated *Enteromorpha* into Fe─N─C SACs with Fe─N_4_─C configuration (**Figure** **1**a) and metal loading exceeding 1.5 wt.%, demonstrating excellent performance in Fenton‐like reactions.^[^ ^56^ ^]^ The increase in urea concentration provided more nitrogen source for coordinating the atomic Fe, resulting in reduced Fe─Fe bonds in Enteromorpha‐derived Fe─N─C SACs (Figure 1b). In contrast, the Fe─N_2_O_2_ coordination and large amounts of Fe─Fe bonds formed by the pyrolysis of urea‐free *Enteromorpha*, showed reduced activity. Thus, the decomposition of urea into nitrogen‐containing species during pyrolysis forms additional coordination sites that prevent metal aggregation. Wang et al. exploited urea‐saturated Auricularia auricular‐judae containing inherent N and Fe for SAC synthesis, which involved pyrolysis up to 1000 °C and subsequent acid leaching. The catalysts had sheet‐like structures and high SSA up to 1125.7 m^2^g^‒1^. The results demonstrated that atomic Fe existed as Fe─N_4_ for oxygen reduction reaction (ORR) and that the catalytic performance of Fe─N─C SACs could be improved by pyrrolic‐N doping.^[^ ^57^ ^]^ The samples prepared without N‐doping had a comparably lower SSA than N‐doped samples.

In some cases, both external dopants and metal salts are needed. Liu et al. ultrasonically dispersed Cu(NO_3_)_2_.3H_2_O in ethanol solution and introduced phenanthroline as both nitrogen source and bidentate ligand when working with α‐cellulose, achieving Cu–N–C SACs featuring a typical cellulose‐like wrinkled surface with a coarse texture, mesoporous structure and a Cu metal loading of 4.08 wt.% suitable for catalytic Fenton‐like reactions.^[^ ^58^ ^]^ The dual role of phenanthroline as N precursor and bidentate ligand facilitated Cu–N anchorage within the cellulose matrix, resulting in a Cu–N_4_ coordination. This dual role represents an efficient approach to maximizing metal dispersion within cellulosic matrices. It is worth noting that the presence of certain Lewis‐acid metal salts, particularly Fe^3+^, serves dual functions in biomass conversion to SACs. Beyond providing metal sources, they hydrolyze cellulose and hemicellulose to create microchannels essential for high SSA development. Zhong et al. demonstrated this effect by pre‐treating wood with FeCl_3_, which partially hydrolyzed polysaccharides before carbonization under Ar‐NH_3_ atmosphere and acid etching to form single‐atom Fe─N─C catalyst.^[^ ^59^ ^]^ The sample achieved a remarkable SSA (1183.23 m^2^g^‒1^), pore volume of 0.86 cm^3^g^‒1^ and micro/mesoporous structure for catalytic ORR but relatively low Fe loading (∼0.8 wt.%). Liu et al. used industrial spent acid (SA) containing Fe^3+^ and HCl to activate lotus seedpod (LS) with a natural hierarchical structure.^[^ ^60^ ^]^ Fe^3+^ acted as a Lewis acid “scissor” to hydrolyze the cellulose chain in LS and form rich nanopores (Figure 1c). The HCl contained within the spent acid removed aggregates. LS was first treated by SA, freeze‐dried and then pyrolyzed in the presence of urea to form Fe─N─C SACs with a high SSA (2465.6 m^2^g^‒1^) compared to the catalyst (966.0 m^2^g^−1^) prepared using FeCl_2_ and HCl and the catalyst (148.6 m^2^g^‒1^) prepared without SA treatment, thus highlighting the beneficial role of Fe^3+^ as a Lewis acid. Moreover, the catalyst prepared without SA treatment exhibited a smooth and compact surface without visible pores, emphasizing the pore‐directing effect of Fe^3+^. Fe atoms were well‐dispersed throughout the carbon matrix (Figure 1d). Fe loading of approximately 0.55 wt.% was attained and the catalyst had a pore volume of 1.426 cm^3^g^‒1^ and a micro‐ and mesoporous structure with surface roughness for ORR application. The combination of micropores and mesopores is reported to be beneficial for electrocatalysis since the mesopores can act as channels for reactant exchange and the micropores can expose the active sites.^[^ ^61^ ^]^ These examples illustrate how Lewis acid metals enhance porosity development while simultaneously serving as precursors for catalytic sites.

To improve the loading of metal atoms, chelating agents or surfactants can be introduced to enhance interaction with metal ions and support. In the double‐anchoring strategy employed by Wang et al., Ethylenediaminetetraacetic acid (EDTA) was used to chelate Mn^2+^ before adsorption onto biomass (semen sterculia lychnopherae) followed by lyophilization.^[^ ^62^ ^]^ The EDTA‐Mn‐biomass was then ground with melamine for two‐stage pyrolysis at 550 and 800 °C, during which Mn atom was captured by N species, producing Mn–N–C SACs with high Mn loading of 7.8 wt.%. The catalyst had a pore volume of 1.48 cm^3^g^−1^, SSA of 123.3 m^2^g^−1^ and exhibited a yarn‐like structure with ultrathin 2D graphitic carbon layers due to the role of melamine in thinning the carbon layer for ozonation application.

Pyrolysis temperature influences both nitrogen retention and graphitization degree, creating a balance for optimal SAC formation. Relatively lower temperatures (500–600 °C) preserve nitrogen content but may limit graphitization degree and pore development. Higher temperatures (800–1000 °C) enhance graphitization and surface area but risk nitrogen loss. Some plants, such as *Phytolacca (P.) americana*, have the potential to accumulate metals of high concentrations. To simulate the utilization of metal‐contaminated plant biomass, metal or dopants were incorporated into the plant by irrigation, and the harvested plant biomass was used as a precursor for direct SAC synthesis. For example, the root of MnCl_2_‐irrigated *P. americana* was employed for the pyrolyzed synthesis of single‐atom Mn immobilized on N‐doped carbon (Mn─N─C SAC).^[^ ^50^ ^]^ Increasing temperature from 550–750 °C improved both SSA (to 470.5 m^2^g^‒1^) and Mn loading (1.13 wt.%) with micropore generation and atomic Mn–N_4_ sites for photocatalytic organic pollutant degradation. Higher annealing temperature can strengthen the interaction between Mn and carbon substrate, for the stabilization and the atomic diffusion of Mn. Similarly, Yang et al. found that step‐wise pyrolysis (to 700 °C) of *P. americana* stem watered by MnCl_2_ or urea strengthened Mn‐carbon matrix interactions, resulting in undetectable Mn leaching compared to significant leaching at 500 °C.^[^ ^63^ ^]^ The carbon matrix tends to possess a more ordered and graphitic structure with increased pyrolysis temperature for effective stabilization of endogenous Mn in the matrix. Li et al.^[^ ^54^ ^]^ used residues of a heavy metal hyperaccumulator plant, Sedum alfredii, sampled from a Zn‐contaminated site and enriched with Fe to synthesize Fe─N─C SAC. The plant material was pyrolyzed at 800 °C for 2 h, facilitating Zn evaporation, leading to the self‐dispersion of Fe atoms and the formation of Fe─N─C SAC with FeN_3_O_1_ sites, responsible for the catalytic activity in Fenton‐like reactions. The catalyst had an optimal Fe loading of 1.41 wt.%, SSA of 25.52 m^2^g^‒1^, and a smooth‐edged sheet structure with wrinkled, graphene‐like layers.

Direct pyrolysis of biomass provides a versatile route for the synthesis of SACs, with success highly dependent on the intrinsic composition of the biomass and pyrolysis parameters. Biomass precursors containing nitrogen and/or metal species can yield atomically dispersed active sites through thermal treatment, although low heteroatom content often limits metal coordination and loading. Strategies to overcome this include incorporating external dopants such as urea, metal salts, or chelating agents to enhance coordination environments and prevent metal aggregation. Additionally, the use of Lewis‐acid metal salts like Fe^3+^ offers dual benefits by acting as both metal precursors and porosity promoters through hydrolysis‐induced microchannel formation. Among the various processing strategies, solution‐based impregnation and mechanical mixing (detailed in Section 2.1.8) offer versatile and scalable approaches for introducing metal species into biomass‐derived matrices. Pyrolysis temperature is a critical factor, influencing both nitrogen retention and graphitic structure. These studies demonstrate that careful control of biomass composition, metal coordination chemistry, and pyrolysis conditions enables the rational design of biomass‐derived SACs with tailored active sites and porosity for advanced catalytic applications.

#### 2.1.2 Mechanical Treatment‐Assisted Biomass Conversion to SACs

Compared with solution‐based synthesis, mechanical treatment approaches are solventless and environmentally friendly synthesis routes^[^ ^64^ ^]^ with low installation and operational costs. Hybrid strategies combining solid‐state mixing with solution‐based impregnation are also employed to enhance precursor interaction and improve metal dispersion. Many aforementioned examples involve solution‐based precursor interaction and grinding, a low‐energy solid‐state mixing method. In contrast, ball milling is a high‐energy mechanical process that facilitates the uniform dispersion of metal species and promotes close contact between precursors through intensive mechanical force. Other approaches like mechanical compression can create a confinement effect for SAC synthesis.

Grinding is often used for initial mixing or simple precursor preparation. For example, Porphrya seaweed powder, rich in proteins and taurine, and a source of S and N was mixed with FeCl_3_ by grinding in mortar.^[^ ^44^ ^]^ Taurine, with its charged amino and carboxyl groups, binds to Fe ion, forming metal‐amino acid complexes. The FeCl_3_‐encapsulated‐porphyra precursor was subjected to an annealing process at 900 °C. During this process, bioactive peptide and taurine provided N, S co‐doped C. Simultaneously, the biocarbon was stripped into thin, porous sheets due to the release of carbon dioxide and nitrogen dioxide gases during pyrolysis. Fe potentially reacted with nearby C, N, and S atoms. Finally, the product was acid‐etched with 2 M HCl at 85 °C for 3 h, creating a porous S‐doped Fe─N─C SACs. Fe–N–S–C SACs showed a thinner stripped flake with porous and wave‐like rippled structure and SSA of 1533.7 m^2^g^−1^. This structure shows advantages for both mass and electron transfer, facilitating maximum effective contact of active single Fe sites in ORR.

Ball milling is often used to achieve uniform precursor mixing and control metal dispersion in fabricating biomass‐derived SACs. As another example, Hou et al. employed a solid‐state method by ball milling Chlorella vulgaris powder with inherent N and FeCl_3_.6H_2_O in a mass ratio of 4:1 before pyrolysis at 800 °C.^[^ ^65^ ^]^ The resulting Fe─N─C SACs featured both single Fe atoms and small clusters with an SSA of 652.29 m^2^g^‒1^, demonstrating that mechanical mixing can effectively distribute metal precursors throughout biomass matrices. However, careful control of metal precursor concentration is important, as excessive FeCl_3_.6H_2_O resulted in Fe aggregation despite mechanical mixing. Zhou et al. utilized spirulina (a protein‐rich biomass with high hydrogenase content) with K_2_CO_3_ activation for the synthesis of Mn─N─C SACs.^[^ ^51^ ^]^ Spirulina and K_2_CO_3_ were dispersed in deionized water, stirred, dried, and carbonized at 400 °C in N_2_. Mn salts and dicyandiamide were dispersed in deionized water, stirred, dried, and carbonized at 550 °C in air. The carbonized products were mixed, then ball‐milled and the resulting precursor was pyrolyzed at 700 °C to form Mn─N─C SACs. The combination of dicyanamide and inherent N content from spirulina creates a strong coordination with Mn salts, forming highly dispersed MnN_4_ sites for efficient Fenton‐like reaction. Xue et al. soaked cotton stalk powder in Fe(NO_3_)_3_ solution, followed by stirring, centrifuging, washing, and drying.^[^ ^66^ ^]^ The resultant powder was uniformly mixed with melamine by ball milling for 10 min at 600 rpm, then the obtained powder was carbonized at 800 °C. Hemicellulose, cellulose, and lignin present in cotton stalk provided rich oxygen functional groups to anchor Fe ions. The lamellar wrinkled morphology with well‐dispersed Fe atoms demonstrated how mechanical treatment with N sources can prevent metal aggregation. The catalyst had a Fe─N_3_O─C configuration with 1.3 wt.% Fe loading and was applied for Fenton‐like reactions. Wu et al. employed a one‐pot strategy combining ball milling of protein‐rich biomass (pig liver powder) with molten salt‐assisted carbonization and obtained Fe─N─C SACs with a winding carbon sheet‐like structure, SSA of 803.1 m^2^ g^‒1^, and metal loading of 2.6 wt.%.^[^ ^42^ ^]^

Taking advantage of the *Myriophyllum aquaticum* herb with its highly porous structure and N element, Li et al. demonstrated the integration of biomass structure (*Myriophyllum aquaticum* herb) with ball milling and activation strategies. Pre‐carbonized *Myriophyllum aquaticum* was ball‐milled with K_2_FeO_4_ (serving dual roles as pore‐directing agent and Fe source).^[^ ^52^ ^]^ Thereafter, the obtained nanoparticles deposited on the hierarchical porous biochar were acid‐etched to produce Fe─N─C SACs with a higher Fe loading (2.4 wt.%), disordered degree, and SSA (2040 m^2^ g^‒1^). The catalyst exhibited a thin carbon layer and hierarchical porous structure for peroxymonosulphate (PMS) activation in Fenton‐like reactions.

Mechanical compression also represents another solid‐state mixing method. Li et al. developed a hybrid approach combining one‐pot hydrothermal pre‐treatment of cellulose with mechanochemical techniques for the synthesis of various metal (M = Fe, Co, Ni, Cu, and Zn) SACs with M−O_3_C coordination.^[^ ^47^ ^]^ The different metal nitrates and cellulose were mixed using ultrasonic treatment in an autoclave and then hydrothermally pre‐treated at 240 °C. Then, the obtained precursor was filtered, washed, dried, and compressed into pellets/disks for pyrolysis at 800 °C, followed by acid etching. Hydrothermal treatment resulted in monodispersed metal ions within the carbon matrix, while mechanical compression created a confinement effect that stabilized single atoms during pyrolysis. This study also demonstrated the synthesis of carbon‐based SACs by utilizing natural oxygen as anchoring sites and carbon as the skeleton in biomass feedstock, achieving metal loadings of 0.29 – 1.15 wt.%, and SSAs of 388–647 m^2^g^−1^, for photocatalytic degradation applications.

To sum up, mechanical solid‐state treatments, ranging from grinding, ball milling, to mechanical compression, play a key role in the green synthesis of biomass‐derived SACs. These methods enable efficient mixing, uniform metal dispersion, and porous structure development, particularly when integrated with tailored pre‐treatments and activation strategies.

#### 2.1.3 Zn Salt‐Assisted Biomass Conversion to SACs

Zn salts play multifunctional roles in biomass conversion to biochar, serving simultaneously as economical mesopore‐directing reagents,^[^ ^67^ ^]^ dehydrating agents, spatial isolators for metal atoms, and sources of Zn metal. Unlike conventional porogens, ZnCl_2_ and Zn(NO_3_)_2_ not only create porous structures but also effectively increase spatial distances between metal atoms during synthesis,^[^ ^68^ ^]^ preventing aggregation and promoting atomic dispersion. This unique combination makes Zn salt‐assisted pyrolysis particularly effective for converting various biomass feedstocks into high‐performance SACs.

**Figure 2:**
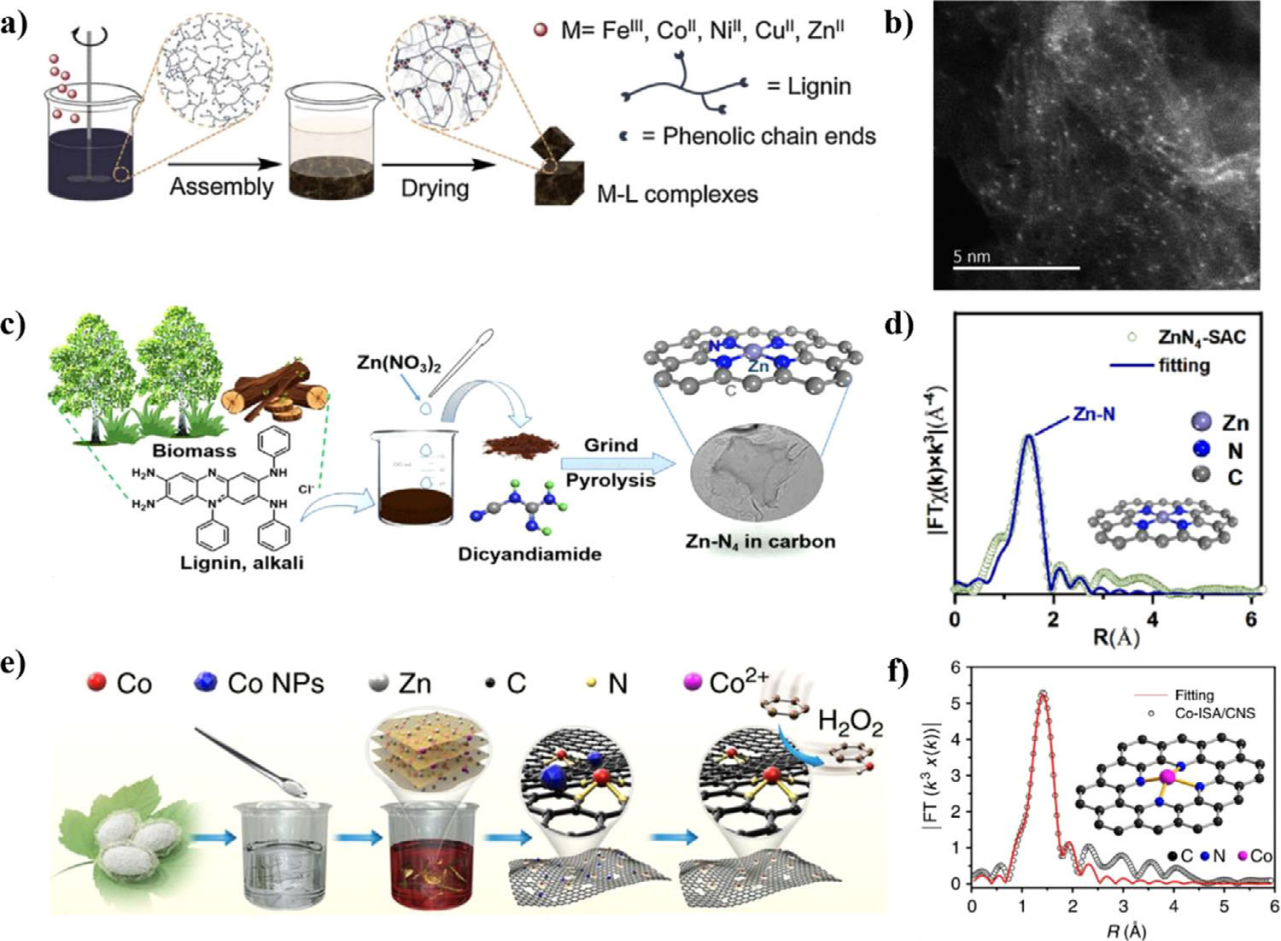
Zn salt assisted conversion of biomass sources. a) Schematic illustration of the assembly process of Co‐lignin complexes from Zn(NO_3_)_2_ salt‐assisted lignin conversion and b) HAADF‐STEM images of Co─N─C SACs. Reproduced with permission.^[^ ^26^ ^]^ Copyright 2019, Elsevier. c) Schematic of the preparation of the Zn─N─C SACs and d) fitted Fourier‐transformed Extended X‐ray absorption near edge structure (FT‐EXAFS) spectra in the R space. Reproduced under terms of the CC‐BY licence.^[^ ^71^ ^]^ Copyright 2023, American Chemical Society. e) Synthesis scheme for Co─N─C SACs from cocoon silk and (f) corresponding EXAFS fitting curves in the R space. Reproduced under terms of the CC‐BY licence.^[^ ^27^ ^]^ Copyright 2018, Springer Nature.

Zn salts have been frequently employed to facilitate the conversion of lignin into SACs. The unique crosslinked phenolic polymer structure of lignin is compatible with salt‐assisted synthesis approaches. Its abundant phenolic hydroxyl groups function as natural chelating sites, forming an encapsulating “spider web” that effectively captures metal atoms for metal‐lignin complexes.^[^ ^26^ ^]^ In a typical process, Zn salt, metal (Fe, Co, Ni, or Cu) salt, and lignin were self‐assembled in water with stirring, and the resulting Zn/metal/lignin complex was then separated via centrifugation and dried (**Figure** **2**a).^[^ ^26^ ^]^ This was followed by mixing with dicyanamide by grinding in a mortar. The activation involved an annealing step at 550 °C, followed by a temperature increase to 1000 °C to allow for the evaporation of Zn, resulting in the formation of single‐atom sites. In this study, Co─N─C SACs were prepared using lignin, Co(NO_3_)_2_·6H_2_O, and Zn(NO_3_)_2_·6H_2_O and dicyandiamide as raw materials. The resulting catalyst exhibited a homogenous disordered carbon structure with 1.54 wt.% Co loading, where atomic cobalt existed in Co─N_3_ sites. This catalyst demonstrated catalytic activity for the oxidative esterification of primary alcohols, with isolated Co atoms observed on the carbon matrix (Figure 2b). By a similar approach, Qi et al. demonstrated control over metal loading by modulating Co salt concentration in lignin‐Zn complexes, increasing Co content from 0.26 to 2.35 wt.% as Co salt rose from 2 to 24 mM.^[^ ^25^ ^]^ However, further increasing Co salt to 40 mM, while enhancing metal loading to 3.90 wt.%, resulted in a small increase in catalytic performance for Fenton‐like reactions due to pore clogging, demonstrating a relationship between metal loading and accessibility of active sites. Peng et al. co‐impregnated lignin with both cobalt and zinc nitrates, followed by controlled aging and drying, then dicyandiamide addition as a nitrogen source before subjecting the mixture to a strategic two‐step pyrolysis to 1000 °C.^[^ ^69^ ^]^ The Zn‐assisted volatilization strategy generated isolated Co atoms anchored predominantly in CoN_3_ coordination environments within a disordered carbon framework. The resulting catalyst exhibited a curly sheet morphology, 1.15 wt.% Co content and SSA of 305.33 m^2^g^‒1^ for Fenton‐like reactions.

Beyond controlling metal loading and dispersion, Zn salts also serve as structural templates, enabling the tuning of metal coordination environments through controlled pyrolysis temperature. Park et al. demonstrated this templating effect by synthesizing Ni─N─C SACs with consistent Ni loading (≈0.43 wt.%) but different coordination structures depending on pyrolysis conditions.^[^ ^70^ ^]^ As temperature increased from 800 to 1000 °C, the dominant coordination environment evolved from Ni─N_4_ to Ni─N_3_C and ultimately Ni─N_2_C, with the unsaturated Ni─N_2_C sites exhibiting optimal activity for carbon dioxide reduction reactions (CO_2_RR). This coordination evolution occurs because higher temperatures promote nitrogen loss from the carbon framework, creating coordination vacancies that carbon atoms subsequently fill. Importantly, the low electronegativity of residual Zn relative to Ni ensures it functions purely as a structural template without interfering with Ni coordination, highlighting the selective role of Zn as primarily a structural template rather than a competing coordinating agent.

In another work, lignin was separated from enzymatic hydrolysis residue of corn stalk, which was dissolved in NaOH solution.^[^ ^40^ ^]^ A solution containing CoCl_2_ and anhydrous ZnCl_2_ was then added dropwise to lignin solution. The phenolic‐(OH)‐rich lignin formed stable Co/Zn‐lignin complex precipitates, which were separated by centrifuging. This study employed a solution‐based approach by mixing a Co/Zn‐lignin complex with melamine in water, followed by freeze‐drying. The resulting mixture underwent two‐step pyrolysis at 550 and 900 °C, along with acid etching, to produce Co─N─C SACs. Introducing Zn atoms helped increase the spatial distance between adjacent Co atoms by partially substituting Co binding sites. As a result, Co─N─C SACs with a fluffy and laminar architecture were synthesized, suitable for use in the reductive fractionation of poplar biomass.

Zn salts also contribute to the formation of single atom Zn sites, which show multiple functions for oxidative cleavage of C─N bonds during organic and biochemical transformations. For instance, lignin alkali and Zn(NO_3_)_2_·6H_2_O were mixed and stirred in solution to form a Zn‐lignin complex, which was subsequently separated, dried, and combined with dicyandiamide through grinding. The two‐step pyrolysis at 550 and 1000 °C produced Zn─N─C SACs (Figure 2c) with Zn─N_4_ coordination sites (Figure 2d),^[^ ^71^ ^]^ 0.67 wt.% Zn loading, pore volume of 0.96 cm^3^g^‒1^ and SSA of 584 m^2^g^‒1^. The single‐atom Zn sites demonstrated catalytic activity for oxidative cleavage of C─N bonds during organic transformations. This dual role, where Zn simultaneously serves as a pore‐forming templating agent and a catalytic metal precursor, represents a unique advantage of Zn‐assisted synthesis over conventional approaches that require separate addition of porogens and metal source.

Zn salts demonstrate versatility across different biomass types beyond lignin. Depending on biomass types, metal salt type, and other precursors, the optimal metal salt amount varies for SAC synthesis. Generally, metal salts, Zn salts, and biomasses are dispersed in solution, and evaporated to obtain the metal/Zn/biomass mixture for pyrolysis. Zhu et al. used silk fibroin extracted from the cocoon in the fabrication of Co─N─C SAC. The obtained mixture of silk fibroin, CoCl_2_, and ZnCl_2_ was pyrolyzed at 900 °C for 1 h, followed by acid etching to form Co─N─C SAC (Figure 2e) with an ultrahigh SSA of 2105 m^2^g^−1^, nearly double the SSA (1061 m^2^g^−1^) of the sample achieved without Zn treatment.^[^ ^27^ ^]^ This enhancement demonstrates the pore‐forming capability of Zn. The catalyst formed had a pore volume of 1.7 cm^3^g^−1^, a uniform sheet‐like 2D structure, Co loading of 0.6 wt.%, and Co─N_4_ configuration (Figure 2f) for the catalytic oxidation of benzene. Similarly, chitosan‐derived Co─N─C SACs achieved a very high SSA of 2513 m^2^g^−1^ and a large pore volume of 2.2 cm^3^g^−1^ through ZnCl_2_‐assisted synthesis.^[^ ^72^ ^]^ However, the significant SSA enhancement came with a trade‐off of a substantially reduced Co loading (0.1 wt.%) compared to 2.6 wt.% without Zn addition. The inverse relationship between surface area and metal loading presents a fundamental challenge in Zn‐assisted synthesis, requiring careful optimization based on application requirements. However, excessive Fe(NO_3_)_3_·9H_2_O salts were found to generate nanoparticles in another study. Jiao et al. used corn silk, Fe(NO_3_)_3_·9H_2_O, ZnCl_2_, and melamine to synthesize Fe─N─C SACs, with Fe loading exceeding 4.3 wt.% and nitrogen content reaching 10 wt.%.^[^ ^73^ ^]^ The Fe─N─C SACs had abundant single‐atom sites for use as an electrode material in both ORR and oxygen evolution reaction (OER) for Zn–air batteries. The resulting 3D hollow structure with multimodal pore distribution (micro‐, meso‐, and macropores) demonstrates how Zn salts can direct the development of hierarchical porosity rather than simply creating uniform pores. However, maintaining appropriate Fe salt concentration is important as excessive amounts lead to metal agglomeration and the formation of nanoparticles.

In summary, Zn salt has multifunctional uses in synthesizing biomass‐derived SACs, serving as a porogen, a source of single‐atom Zn, and isolating atoms to promote dispersion of other metal atoms. In practical synthesis applications, the optimal structure of SACs requires tailored approaches based on the characteristics of biomass. The appropriate mixing method should be chosen based on the different types of biomass, dopants, the mass ratios of Zn salts, and other metal salts. The appropriate amount of Zn salt is important as excessive Zn salt led to high Zn evaporation and a collapse of the porous structure while insufficient amounts resulted in low evaporation. Besides, low‐concentration metal salts may result in low single‐atom load, while high‐concentration metal salts may result in nanoparticle formation.

#### 2.1.4 NH_4_Cl Activation‐Assisted Biomass Conversion to SACs

NH_4_Cl serves as both a nitrogen source and a pore‐forming agent during biomass pyrolysis. When heated, NH_4_Cl starts to decompose at approximately 340 °C, releasing NH_3_ and HCl gases that introduce N atoms into the carbon framework and generate microporous structures.^[^ ^74^ ^]^ Wang et al. demonstrated this approach by preparing a transparent solution containing chitosan, gas foaming agent (NH_4_Cl salt), Co(Ac)_2_·4H_2_O and acetic acid in water.^[^ ^75^ ^]^ After freeze drying and pyrolysis at 850 °C, followed by HCl pickling to remove excess cobalt, they obtained Co─N─C SACs with 1.27 wt.% Co loading in the form of Co─N_4_ sites. The success of this method stems from the unique properties of chitosan. Its amino groups show cationic behavior in acidic conditions, creating electrostatic interactions with metal ions^[^ ^76^ ^]^ that prevent aggregation and promote atomic dispersion.^[^ ^77^ ^]^ Meanwhile, NH_4_Cl decomposition creates a hierarchical 3D porous network with ultra‐thin nanosheet morphology, affording a high SSA of 1977.9 m^2^ g^‒1^ and pore volume of 2.99 m^3^ g^‒1^. By comparison, only a few pores were observed on the catalyst surface prepared without NH_4_Cl. The carbonization temperature modulated the structure and morphology of the catalysts, with the thickness gradually decreasing as the temperature increased from 750 to 850 °C. However, at higher temperatures (900 °C), a subtle structural collapse was observed, reducing SSA and the number of pores. This example highlights the synergistic structure‐activity relationship where precursor choice, activation strategy, and synthesis temperature synergistically influence catalyst properties. The formation of highly dispersed single sites and hierarchical porosity is important for high catalytic activity. Compared to non‐NH_4_Cl‐assisted methods, the NH_4_Cl pathway shows better control over metal dispersion and porosity.

#### 2.1.5 Molten Salt‐Assisted Biomass Conversion to SACs

**Figure 3:**
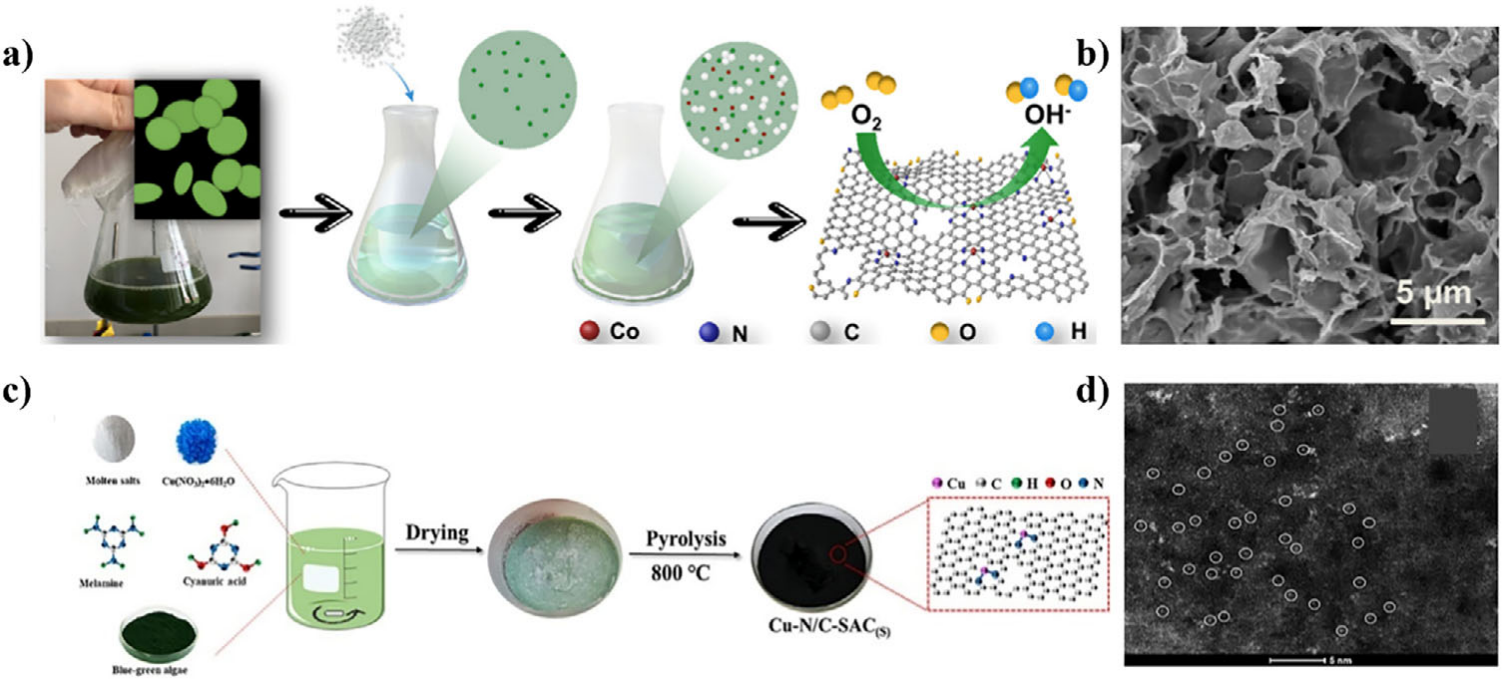
Molten‐salt assisted synthesis strategies of biomass‐derived catalysts. a) Synthesis scheme for Co─N─C SACs and (b) corresponding SEM image. Reproduced with permission.^[^ ^83^ ^]^ Copyright 2021, Elsevier. (c) Synthesis scheme and corresponding (d) AC‐HAADF‐STEM image for Cu─N─C SACs. Reproduced with permission.^[^ ^84^ ^]^ Copyright 2024, Elsevier.

Molten salt‐assisted SAC synthesis uses molten inorganic salts such as LiCl, KCl, and NaCl^[^ ^78^ ^]^ as templates, stabilizers, and reaction mediums. During pyrolysis at high temperatures, molten salt forms a liquid state, serving as a solvent or template for the formation of porous structures.^[^ ^79^, ^80^ ^]^ Meanwhile, the free anions and cations in the molten salt provide high polarity,^[^ ^81^ ^]^ which breaks chemical bonds and prevents aggregation of metal precursors, promoting the formation of uniformly dispersed single‐atom catalysts. Molten salt can act as a pore‐forming agent during carbonization,^[^ ^82^ ^]^ enhancing the surface areas of biomass‐derived catalysts. By this approach, Wu et al. mixed chlorella vulgaris with rich amino groups, Co(NO_3_)_2_·6H_2_O, and NaCl/KCl (melting point of 658 °C; the total weight is ten times that of cobalt salt) in water (**Figure** **3**a).^[^ ^83^ ^]^ This was followed by freeze drying, pyrolysis at 800 °C, and water washing to remove the soluble salts to obtain Co─N─C SACs. The catalyst showed a crumpled sheet‐like structure (Figure 3b) with an ultrahigh SSA of 2907 m^2^ g^‒1^, pore volume of 6.3 cm^3^ g^‒1^ and high metal loading of 3.1 wt.%, showing excellent electrocatalytic activity and stability for ORR. The catalyst also contained atomically dispersed Co─N_4_ coordination sites, showing that the interaction between Co^2+^ ions and *Chlorella* biomass is essential for establishing Co─N coordination sites during pyrolysis. Also, the molten salts during high‐temperature pyrolysis serve as a high‐polarity solvent during carbonization process, facilitating the firm anchoring of isolated Co atoms within the carbon matrix while preventing aggregation. Finally, the rational selection of biomass precursors must ensure compatibility with the molten salts environment to achieve optimal single‐atom dispersion. The study shows that molten‐salt activation has a pronounced role in producing carbon‐based SACs with high SSA and metal load. However, for large‐scale development, this strategy faces a challenge due to the high cost of some molten salts.

Cu─N─C SACs were similarly synthesized from blue‐green algae using molten salt‐assisted pyrolysis (Figure 3c).^[^ ^84^ ^]^ The synthesis involved sequential steps: blue‐green algae were first dried and ground into powder while Cu(NO_3_)_2_.3H_2_O and melamine were dissolved in ultrapure water and ultrasonically dispersed at 50 °C for 2 h. NaCl/KCl salts (mass ratio = 1:1) were then added and stirred until complete dissolution, followed by the addition of blue‐green algae and cyanuric acid. After drying and milling, the mixture underwent pyrolysis at 800 °C for 2 h. Cu─N─C SACs were obtained after cooling to room temperature, centrifugation, and washing to remove residual molten salts and drying. The molten salt treatment significantly enhanced the textural properties of the catalyst. The SSA of the catalyst increased from 270.2 to 565.1 m^2^ g^‒1^ after Cu incorporation, while the pore volume of the catalyst decreased slightly from 41.5 to 35.2 nm. Structural characterization confirmed a hierarchical pore structure with irregular morphology. Aberration Corrected (AC) HAADF‐STEM images (Figure 3d) revealed uniform distribution of isolated Cu atoms on biochar, confirming successful single‐atom dispersion suitable for Fenton‐like reactions.

Molten salt‐assisted pyrolysis provides a high‐polarity, liquid‐phase medium that enhances carbon structure development, prevents metal aggregation, and promotes the formation of atomically dispersed metal sites. This strategy enables the synthesis of SACs with high surface area, large pore volume, and well‐defined metal site configurations from biomass such as chlorella and algae. However, the economic cost of molten salts may limit large‐scale application.

#### 2.1.6 Hard Template‐Assisted Biomass Conversion to SACs

Hard templates allow for precise control over pore size, SSA, and structure, which can enhance catalytic activity by providing more accessible active sites. Also, the confined spaces of hard templates (e.g., mesoporous silica) provide an isolated environment and spatial confinement effect that help prevent metal atoms from aggregating, promoting the formation of stable single‐atom catalysts.^[^ ^8^, ^11^ ^]^ In this case, biomass should be sufficiently dispersed in a solution or medium to ensure even mixing and interaction with the metal precursor and the hard template. The existence of functional groups (like amino, hydroxyl, or carboxyl groups) is also desired to chelate metal ions and uniformly distribute them. This coordination chemistry determines the resulting metal‐nitrogen configuration in the final SAC structure.

**Figure 4:**
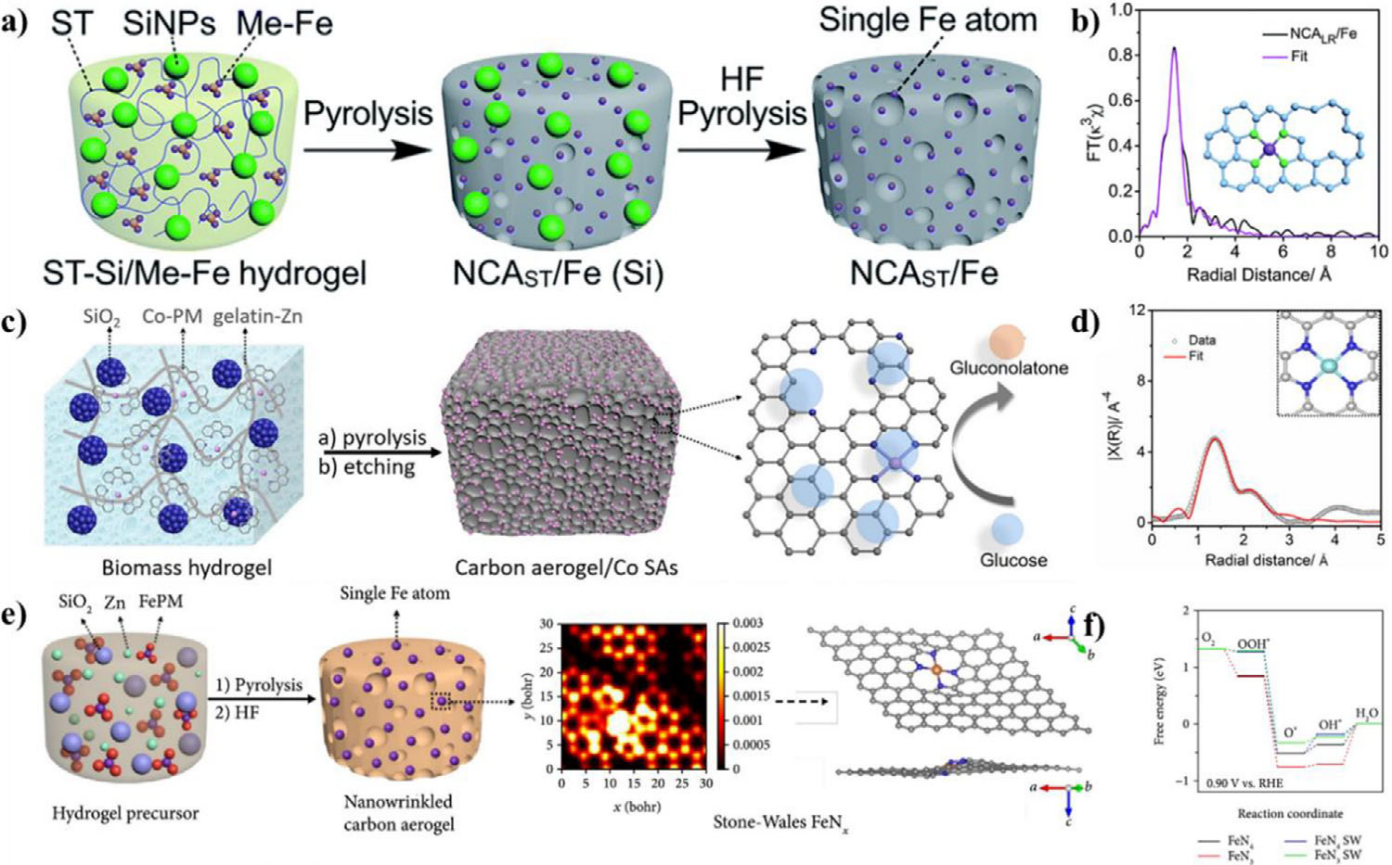
Synthesis strategies for biomass‐derived SACs using template‐assisted approaches. (a) Illustration of the fabrication procedure of Fe─N─C SACs from lotus root and (b) corresponding EXAFS and fitting curve. (Inset: structure of FeN_4_ within a defective carbon substrate). Reproduced with permission.^[^ ^41^ ^]^ Copyright 2019, Royal Society of Chemistry. (c) Illustration of the preparation of Co─N─C SACs from gelatin and (d) corresponding EXAFS spectrum and fitting curve. (Inset: schematic of the CoN_4_ site). Reproduced with permission.^[^ ^48^ ^]^ Copyright 2022, Elsevier. (e) Synthesis scheme of Fe─N─C SACs derived from chitosan. (f) Free energy diagrams of ORR processes on FeN_4_, FeN_3_, FeN_4_ SW, and FeN_3_ SW. Reproduced under terms of the CC‐BY licence.^[^ ^23^ ^]^ Copyright 2019, American Association for the Advancement of Science.

Hard templates are mostly inorganic‐based materials such as silica for the development of nanopores. He et al. demonstrated the synergistic combination of natural biomass structure with hard templates using lotus root as a precursor for Fe─N─C SACs aerogel.^[^ ^41^ ^]^ Powdered lotus root, FeCl_2_, and SiO_2_ nanoparticles were dispersed in melamine solution in boiling water by ultrasonication to form hydrogel, followed by freeze drying, pyrolysis at 850 °C, HF etching, and a second pyrolytic treatment at 950 °C (**Figure** **4**a). The cross‐linked structure of lotus roots provided a natural hierarchical structure that created a network of pores for distributing active sites and allowed for tuning the structure of the hydrogel to increase microporous structures. The catalyst showed monodispersed Fe–N_4_ active sites (Figure 4b), a honeycomb‐like hierarchical porous structure, SSA of 699.8 m^2^ g^‒1^ and 1.3 wt.% Fe loading for ORR. A 20‐fold higher Fe loading compared to melamine‐free synthesis highlights the role of nitrogen in trapping and stabilizing single‐atom Fe within carbon aerogels. As another example, Song et al. used gelatin to fabricate carbon aerogels with Co single atoms, in the presence of SiO_2_ template, Co(Ac)_2_·4H_2_O/1,10‐phenanthroline monohydrate‐formed metal complex and  Zn(Ac)_2_, which were suitably mixed to form a hydrogel (Figure 4c), followed by freeze drying, pyrolysis in 3% H_2_ at 900 °C and acid etching.^[^ ^48^ ^]^ The 3D structure of nitrogen‐doped carbon aerogels facilitated the dispersion of Co single atoms and mass transfer during electrochemical detection of glucose by oxidation. The catalyst had an SSA of 827.2 m^2^ g^‒1^ and exhibited microporous defects and nano‐wrinkles with a Co loading of 1.2 wt.%. EXAFS analysis indicated a mixture of Co–N_4_ and Co–N_3_ coordination environments (Figure 4d). In another approach, Wu et al. used a biomass‐assisted pyrolysis‐etching‐activation strategy to immobilize various single‐atom metals (Co, Fe, or Ni) on N, B co‐doped porous carbon.^[^ ^85^ ^]^ Precursors including starch, SiO_2_, CoCl_2_·6H_2_O, dicyandiamide, and boric acid were mixed in solution and steam‐evaporated, followed by pyrolysis, etching, and H_2_ activation. With an SSA of 638.3 m^2^g^‒1^, 1.9 wt.% Co loading, and atomic Co–N_3_–B sites, the obtained Co─N─B─C catalyst had a wide range of catalytic applications, including O‐silylation, oxidative dehydrogenation, and transfer hydrogenation.

Hard template‐assisted synthesis has significant benefits in terms of metal dispersion, and stability of single‐atom metals. The pores provided by hard templates help provide confinement effects to promote single‐atom metal site formation.^[^ ^8^ ^]^ Further, the addition of a carbon precursor with a natural hierarchical structure supports better atom anchorage. The porosity of the carbon support can be tuned by controlling the amount of silica.^[^ ^86^ ^]^ However, challenges such as silica template removal by hazardous HF etching,^[^ ^87^ ^]^ scalability limitations, and environmental impact must be addressed for practical applications.

#### 2.1.7 Soft Template‐Assisted Biomass Conversion to SACs

Soft templates, primarily organic‐based materials such as block copolymers and surfactants, are used to construct porous architectures. These templates are typically removed during the pyrolysis process. Soft templates can be broadly classified into externally sourced templates and self‐templates, offering precise control over pore size and distribution, enabling uniform dispersion of metal atoms and high SSAs.

P123, a copolymer, facilitates the formation of a mesoporous structure during calcination. Li et al. used waste ferns inherently containing iron from mining operations with P123 as a template.^[^ ^88^ ^]^ The waste ferns were ground with ethanol after which NH_3_.H_2_O was added, and the mixture ultrasonically dispersed for 1 h. The dissolved plant was transferred to a solution of P123 in ethanol, sonicated and mechanically stirred for 1 h. The obtained gel was centrifuged and dried before pyrolysis at various temperatures up to 1000 °C followed by acid etching to synthesize Fe─N─C SACs. The catalyst pyrolyzed at 800 °C exhibited the highest SSA of 275.6 m^2^ g^‒1^ and a predominance of mesopores (14.97 nm) over micropores (1.03 nm), originating from P123, with Fe atoms present as Fe–N_4_ sites for photocatalysis. However, exposure to temperatures above 800 °C resulted in a collapse of the pore structures due to pore shrinking. This temperature‐dependent structural change establishes a synthesis limitation for P123‐templated systems. In contrast, Mai et al. employed a one‐step hydrothermal pre‐treatment of chitosan using F127 as a surfactant.^[^ ^89^ ^]^ A hydrogel formed from a mixture of chitosan dissolved in ethanol, F127, 4‐aminophenol, hexamethylenetetramine (HMT), acetic acid, and Co salt was hydrothermally treated at 100 °C for 12 h in an autoclave, freeze‐dried, and then calcined at 800 °C, followed by acid‐etching. The resulting catalyst had a pore volume of 0.504 cm^3^ g^‒1^. The surfactant introduced during pyrolysis enhanced the mechanical properties and functioned as a soft template to produce mesopores, micropores, and a 3D cross‐linked coral structure with an SSA of 371.75 m^2^ g^‒1^, suitable for electrocatalytic ORR applications. In comparison, the catalyst prepared without F127 exhibited a low SSA (80.89 m^2^ g^‒1^) and pore volume (0.407 cm^3^ g^‒1^). This was attributed to the protective role of F127, which prevented the collapse of the carbon aerogel pore structure during the high‐temperature pyrolysis of the phenolic resin/Co^2+^ complex hydrogel precursor and its role as a soft template in creating porous structures. Biomass selection (chitosan with NH_2_, –OH, and –COOH functional groups) enabled effective chelation of Co atoms, leading to a high loading of 1.88 wt.%.

Some natural biomass polymers, such as chitosan and sodium alginate, serve as effective self‐templates in the construction of porous carbons. Hao et al. used sodium alginate, a co‐polymer derived from seaweed, to synthesize Fe─N─C SACs with high SSA (1364 m^2^ g^‒1^), though with a more moderate Fe loading (0.40 at.%).^[^ ^90^ ^]^ The process involved a mixture of sodium alginate, melamine, and an Fe precursor to form a hydrogel. Sodium alginate was added to an aqueous solution and stirred until fully dissolved. To this solution, FeCl_3_ dissolved in water was slowly added with continual stirring to form a brown‐red hydrogel. Melamine was then introduced into the hydrogel and the mixture was stirred to obtain a homogeneous solution. The hydrogel was then freeze‐dried to form an aerogel. Subsequent pyrolysis at 900 °C and acid leaching produced a catalyst featuring a layered microporous structure with Fe–N_4_ coordination sites for ORR. He et al. demonstrated a more sophisticated approach combining chitosan self‐templating with Fe phenanthroline and Zn salt as porogen.^[^ ^23^ ^]^ After sonication to form a hydrogel, the mixture was freeze‐dried and pyrolyzed, producing Fe─N─C SACs with a nano‐wrinkled mesoporous structure, a pore size of 10 nm, an SSA of 609 m^2^ g^‒1^, and an Fe loading of 0.72 wt.% (Figure 4e). The atomic Fe existed as Fe─N_4_ sites. In contrast, catalysts prepared without phenanthroline treatment or both Zn and phenanthroline treatments showed lower Fe loadings of 0.61 and 0.22 wt.%, respectively. This discrepancy highlights the crucial roles of phenanthroline as a chelating agent and Zn^2^⁺ ions as porogen and gel initiator in embedding Fe centers within the carbon matrix. First‐principles calculation revealed that FeN_x_ sites (FeN_4_ SW and FeN_3_ SW) found in the Stone‐Wales (SW) defect created by carbon nanowrinkles have a lower free energy than their typical counterparts (FeN_4_ and FeN_3_) (Figure 4f), increasing the electrocatalytic activity. Thus, the creation of structural distortions of metal sites in carbon matrices is an effective design and engineering strategy for advanced SAC electrocatalysts.

Overall, soft templating enables control over pore structures, ensuring uniform dispersion of metal atoms. While it has significant advantages such as enhanced catalytic performance and functional group integration, challenges like template removal, complex synthesis procedures, and raw material compatibility must be carefully managed.

#### 2.1.8 Microwave‐Assisted Biomass Conversion to SACs

Microwave heating represents a different approach to biomass conversion for SAC synthesis, offering unique advantages over conventional pyrolysis including shorter processing times,^[^ ^91^ ^]^ and the generation of localized “hotspots” with higher heating capacity.^[^ ^92^ ^]^ These hotspots create unique synthesis conditions that influence both structural development and atomic dispersion. As an example, Tian et al.^[^ ^93^ ^]^ demonstrated a microwave approach using Fe‐rich *Enteromorpha* to synthesize Fe─N─C SACs. Specifically, *Enteromorpha* was microwave‐heated at 400 °C. The obtained product was mixed with potassium hydroxide (KOH) in water, followed by drying and microwave‐pyrolysis at 800 °C. KOH activation and high‐temperature hotspots generated during microwave heating led to the formation of a hierarchical porous structure with abundant surface functional groups, defective structures, Fe single atoms, and a large SSA of 1176.5 m^2^ g^‒1^. The surface areas were dependent on temperature and heating time. Catalysts pyrolyzed at 700 °C had relatively lower SSAs (up to 694.7 m^2^ g^‒1^) due to energy limitations at this temperature, leading to partial carbonization and formation of incomplete pores. The microwave‐prepared catalysts showed higher catalytic performance for Fenton‐like reactions than the conventionally heated catalysts. This indicates that the microwave “hotspot” effect enhances the catalytic properties of the catalyst, guiding the synthesis of SACs using an efficient microwave‐initiated approach. While limited studies exist on microwave‐assisted biomass conversion to SACs, this approach demonstrates significant potential for sustainable catalyst synthesis, particularly for biomass precursors with naturally occurring metal content.

In summary, pyrolysis serves as the fundamental approach for the synthesis of biomass‐derived SACs. Depending on the nature of the biomass, SACs can be obtained either through direct pyrolysis or by introducing external metal salts and heteroatom dopants. Additional strategies such as mechanical treatment, chemical activation, or templating methods further enhance the dispersion of metal atoms, control the coordination environment, and tailor the porosity and surface area of the resulting carbon matrix. These pyrolysis‐based routes provide versatile and tunable platforms for the design of SACs with high catalytic performance.

### 2.2 Biomass‐Derived Carbon Supports for SACs Synthesis

Instead of being used directly for SAC synthesis, biomass can be converted into carbon first by pyrolysis or hydrothermal treatment. These processes are often accompanied by an activation process to create porous carbon materials. Porous carbon serves as an excellent support for single metal atoms, due to a high surface area, and various functional groups. These functional groups within the engineered pores stabilize the coordination environment of metal atoms and prevent their aggregation during SAC formation.^[^ ^94^ ^]^

**Figure 5:**
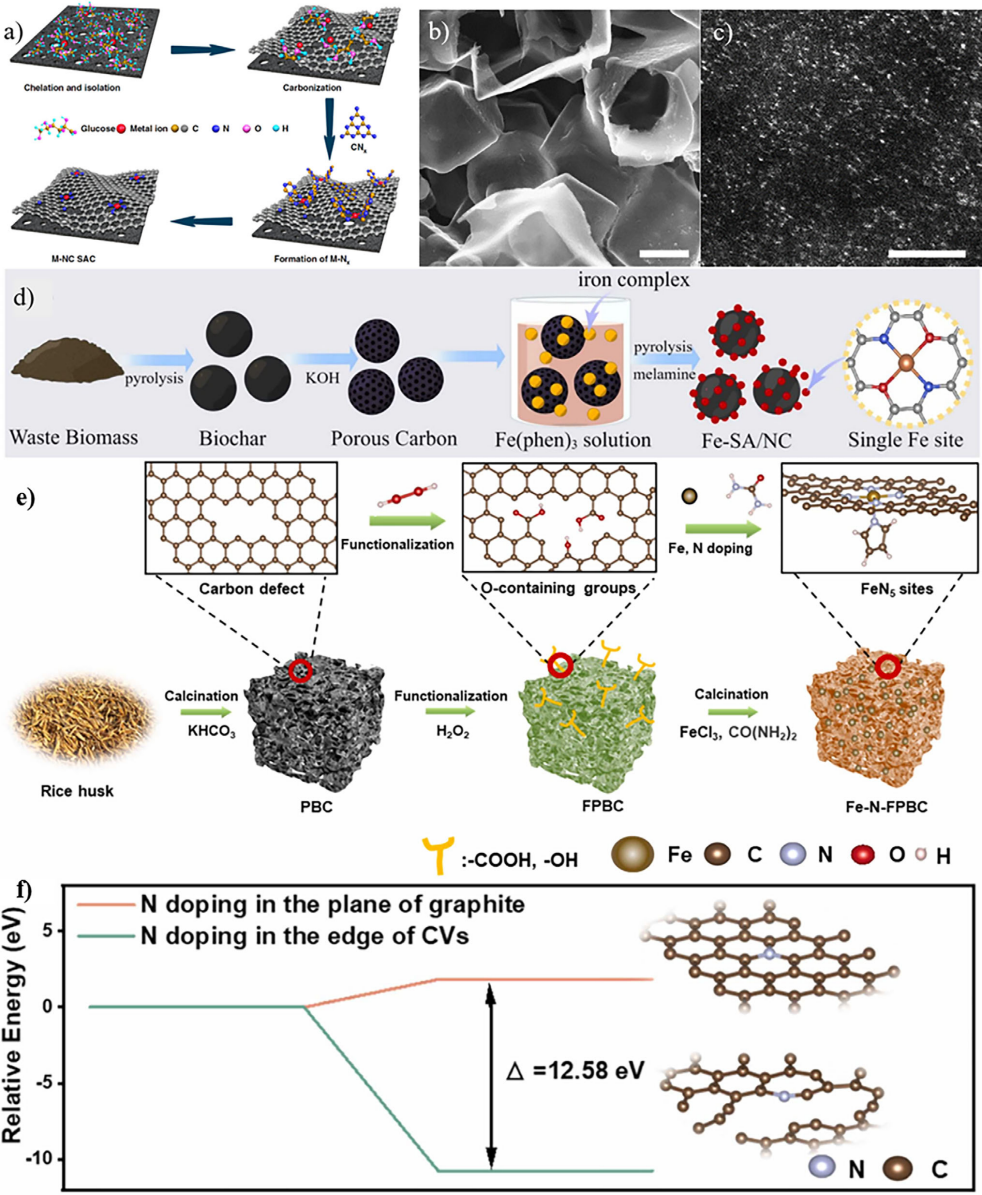
Fabrication of biomass‐derived carbon as support materials for SACs. a) Cascade anchoring strategy for the synthesis of Fe─N─C SACs and corresponding b) SEM image of porous carbon support (scale bar 1 µm) and c) HAADF–STEM image (scale bar 3 nm) of Fe─N─C SAC. Reproduced under terms of the CC‐BY licence.^[^ ^95^ ^]^ Copyright 2019, Nature Publishing Group. (d) Fabrication scheme of Fe─N─C SACs from waste biomass. Reproduced with permission.^[^ ^104^ ^]^ Copyright 2023, Elsevier. (e) Synthesis procedure of Fe─N─C SACs with FeN_5_ sites from rice husk and (f) Relative free energy of N‐doping in the edge of carbon vacancies (inset: model and plane of graphite model). Reproduced with permission.^[^ ^110^ ^]^ Copyright 2024, Elsevier.

The effectiveness of biomass‐derived carbon supports for SACs synthesis depends on three factors, i.e, effective metal ion chelation, spatial isolation of chelated complexes at high loadings, and optimal coordination with heteroatoms at elevated temperatures. Zhao et al. demonstrated the significance of these factors through their cascade anchoring strategy, achieving metal loadings up to 12.1 wt.% across multiple metals (Fe, Co, Mn, Ni, Cu, Pt, Mo) in MN_x_‐porous carbon SACs (**Figure** **5**a).^[^ ^95^ ^]^ This highlights how systematic design approaches can overcome traditional loading limitations while maintaining atomic dispersion. Take Fe‐based SAC as an example, a porous carbon (PC) support with a 3D honeycomb‐like morphology, abundant macropores (Figure 5b), an SSA of 1713 m^2^g^−1^ and rich O species (10 at.%) was ultrasonically dispersed in a solution containing inorganic iron nitrate and α‐D‐glucose (chelating agent), forming a glucose‐Fe (III) complex on the PC surface. The dried mixture was then ground with melamine and pyrolyzed at 800 °C to create Fe─N─C SACs. The choice of iron salt is also crucial. If inorganic iron nitrate is replaced with iron acetylacetonate, the strong interaction between Fe ions and acetylacetone prevents the chelation of glucose with Fe ions. This substitution leads to the formation of numerous iron carbide nanoparticles rather than isolated Fe atoms. Commercial Ketjen black (KB), having a slightly similar SSA of 1400 m^2^g^−1^, can serve as an alternative to PC support, yielding a comparable catalyst structure. Importantly, acid pre‐treatment of KB was essential to generate O‐rich surface, ensuring the effective isolation of Fe centers on the substrate (Figure 5c).

Different biomass sources show unique compositional advantages that can be strategically exploited for enhanced SAC performance. Taking advantage of the high silicon content in rice husk, which is conducive for the formation of oxygen‐containing functional groups on the surface of biochar, Fang et al. used this biomass precursor for the synthesis of Fe─N─C SACs for Fenton‐like reactions.^[^ ^96^ ^]^ Rice husk was crushed and sieved and heated at 800 °C for 2 h. After heating, it was washed with HCl and deionized water followed by drying at 80 °C for 12 h. To synthesize the Fe─N─C SACs, alkaline lignin was mixed with FeCl_2_ and Zn(NO_3_)_2_. 6H_2_O in water and stirred for 1 h to allow coordination of functional groups in lignin with metal ions. The resulting mixture was dried and ground with dicyandiamide before being calcined at 550 °C for 1 h and then 950 °C for an additional hour. Finally, the catalyst was washed with 5 M H_2_SO_4_ and then with water before being dried. The catalyst had a curled lamellar morphology and demonstrated SSA of 156.98 m^2^ g^−1^ and a pore volume of 0.32 cm^3^ g^−1^ to allow for adsorption and transfer of micropollutants in Fenton‐like reactions. Extending this biomass‐specific approach, Gu et al. exploited the mineral salt enrichment in rice straw to develop a ligand‐mediated synthesis strategy for multiple M─N─C SACs (M = Mn, Fe, Co, and Ni).^[^ ^97^ ^]^ Their systematic investigation of cobalt loading effects using different doses of cobalt (II) acetate, i.e 0.1, 0.25, 0.5, and 2.0 mM revealed an optimal metal content of 0.57 at.% using 0.5 mM cobalt (II) acetate, demonstrating how biomass composition can be matched with synthesis parameters to achieve targeted metal loadings. The catalyst also revealed a distinct lumpy structure and was applied for Fenton‐like reactions.

Integrating external heteroatoms with biomass‐derived carbons facilitates the formation of single‐atom sites. Xiao et al. developed an effective two‐step calcination method for synthesizing Co─N─C SACs derived from walnut shells with controlled nitrogen doping.^[^ ^98^ ^]^ The synthesis process involved initial preparation of biochar by crushing walnut shells into fine powder followed by calcination at 700 °C for 8 h in a muffle furnace. The resulting biochar was immersed in Co(NO_3_)_2_ solution and stirred for 24 h, allowing for optimal adsorption‐desorption equilibrium. Subsequently, the biochar was filtered, dried, and combined with dicyanamide as an additional N source. The mixture was carbonized at 550 °C for 6 h and further heated to 1000 °C. The resulting material was pickled with 1 M H_2_SO_4_ for 12 h to remove Co nanoparticles, washed with deionized water until neutral pH was achieved and dried in a vacuum oven at 60 °C to form Co─N─C SACs. The catalyst had a smooth and amorphous structure, total pore volume of 0.0036 cm^3^ g^−1^ and SSA was enhanced by external N addition, achieving improved properties compared to undoped systems. However, the lower SSA (41.92 m^2^ g^−1^) of the optimal catalyst compared to the reference catalyst was attributed to biochar matrix collapse during melting and rearrangement processes. Moreover, the preparation conditions determined the formation of either single atoms or nanoparticles. Shorter residence times at 550 °C produced mixed single atoms and nanoparticles, while the absence of external nitrogen doping resulted in nanoparticle formation, establishing external N‐doping and controlled thermal treatment as important parameters for achieving atomic dispersion.

Suitable biomass selection can eliminate the need for external dopants while achieving desirable properties for SACs. By utilizing the intrinsic dual‐metal and heteroatom content of legume root nodules, Hao et al. developed FeMo─N─C SACs. Specifically, legume root nodules consist of bimetal (Fe, Mo) and heteroatoms (N, S). Pre‐carbonized root nodules at 350 °C were mixed with ZnCl_2_ by grinding and then pyrolyzed up to 1000 °C, facilitating a higher defective structure and SSA (1835 m^2^ g^‒1^).^[^ ^49^ ^]^

Recent advances in biomass conversion have introduced multi‐step approaches to improve the catalyst structure. Liu et al. exploited a three‐step process combining external nitrogen doping, ball milling and pyrolysis to create Co─N─C SACs with a layered structure and well‐dispersed Co single atoms for Fenton‐like reactions.^[^ ^99^ ^]^ Their approach involved separate synthesis of Co‐doped polymerized carbon nitride followed by mechanical mixing with corncob‐derived biochar, enabling independent optimization of each component before final integration. The mixtures were then heated to 700 °C for 2 h. The obtained products were then soaked in 1 M H_2_SO_4_ for 12 h, rinsed with deionized water and ethanol, and vacuum dried at 60 °C. The catalyst exhibited a mesoporous structure despite a relatively low SSA (17.22 m^2^ g^−1^).

Numerous examples have been reported for using biomass‐derived porous carbon support for SAC synthesis. Various methods have been reported for the engineering of porous carbon materials.^[^ ^67^, ^100^ ^]^ KOH is commonly used as an activating agent to generate porous carbons, mainly micropores, from biomass precursors,^[^ ^67^, ^101^ ^]^ which can lead to the creation of high‐surface area carbon supports. Zhang et al. demonstrated this through their multi‐step synthesis using porphyra powder and obtained high SSA (1449.1 m^2^ g^−1^) and Fe loading (2.3 wt.%) through sequential carbonization, KOH activation, and hemin adsorption.^[^ ^94^ ^]^ Tian et al. showed that KOH activation of chestnut shells could produce surface areas reaching 1830.92 m^2^ g^−1^, though subsequent iron phthalocyanine (FePc) introduction reduced these values, indicating potential trade‐offs between surface area and metal loading.^[^ ^102^ ^]^ The influence of KOH activation was further exemplified by the synthesis of Fe‐porphyrin‐based activated carbon that involved a two‐step solvothermal approach, creating a catalyst with honeycomb‐like structure and ultra‐high surface area.^[^ ^103^ ^]^ Initially, divanillin and pyrrole were combined with FeCl_3_⋅6H_2_O in acetic acid and heated at 180 °C for 72 h to form precursors, which were then subjected to KOH activation at 800 °C under nitrogen atmosphere followed by HCl etching. KOH activation transformed the precursor from a low SSA (41.2 m^2 ⋅^g^‒1^) into Fe─N─C SACs with very high SSA (2612.7 m^2^ g^‒1^) and hierarchical porosity (0.8‐16 nm pore diameter). Wang et al. further advanced KOH activation by combining it with mechanical (ball milling) strategies to convert furfural residue (or corn straw, biogas residue) into Fe–N–O–C SACs for Fenton‐like reaction (Figure 5d).^[^ ^104^ ^]^ Furfural residue powder was pre‐carbonized at 600 °C in a muffle furnace to obtain biochar, which was ball milled with KOH, followed by pyrolysis at 800 °C in N_2_ to obtain porous carbon with SSA of 1661.3 m^2^ g^‒1^. Iron acetate was then mixed with phenanthroline to form a Fe(phen)_3_ complex, which was adsorbed by the porous carbon in ethanol solution, followed by evaporation. The resulting solid was mixed with melamine by grinding for pyrolysis in N_2_ to form Fe─N─O─C SACs. Based on the EXAFS fitting results, the single iron atoms existed as Fe–N_2_–O_2_ and were the active sites for PMS activation in Fenton‐like reactions.

Strong alkali activation using KOH can be corrosive, hazardous, and expensive.^[^ ^105^, ^106^ ^]^ This presents significant practical limitations for large‐scale applications, necessitating alternative approaches. Alkali carbonates such as K_2_CO_3_ and NaHCO_3_ are non‐hazardous, inexpensive,^[^ ^107^ ^]^ and have been used as alternative activation agents for carbon‐based materials.^[^ ^67^, ^100^, ^108^ ^]^ Wang et al. prepared porous carbon substrate by pyrolysis of *Enteromorpha* with NaHCO_3_ at 800 °C.^[^ ^109^ ^]^ Then, Fe ions were chelated by glucose, and anchored onto O‐rich porous carbon substrate, and thereafter mixed with melamine by grinding and pyrolyzed at 800 °C to obtain Fe─N─C SACs. The binding of O species in porous carbon with the chelating agent prevented the aggregation of Fe atoms during pyrolysis. The thermal annealing in the presence of melamine was beneficial for the dispersion of Fe atoms, leading to 1.3 wt.% atomic Fe as Fe–N_4_ sites, a mesoporous laminate architecture and SSA of 609.1 m^2^g^−1^ for Fenton‐like reactions. While these values are lower than KOH‐activated catalysts, the non‐hazardous nature and lower cost of carbonate activators make this approach more suitable for industrial implementation.

Du et al. advanced alternative activation through use of KHCO_3_ as a porogen combined with H_2_O_2_ oxidation for functional group introduction in several steps (Figure 5e).^[^ ^110^ ^]^ First, KHCO_3_ was employed to convert rice husks into porous biochar with numerous micropores and carbon vacancies by pyrolysis. Next, H_2_O_2_ was used as an oxidizing agent to introduce additional O functional groups on the surface, with enhanced binding sites for Fe^3+^. Finally, Fe^3+^‐anchored functionalized porous biochar was thoroughly mixed with urea and subsequently pyrolyzed at 800 °C to synthesize Fe─N─C SACs, which featured FeN_5_ coordination structure, 1.28 wt.% Fe, and an SSA of 1023.87 m^2^g^−1^ for catalytic Fenton‐like reactions. Density Functional Theory (DFT) calculations revealed that the energy barrier for nitrogen doping was significantly reduced by biochar with abundant carbon vacancy edges (Figure 5f).

Hydrothermal treatment offers advantages for biomass conversion by retaining high densities of O functional groups,^[^ ^111^, ^112^ ^]^ while eliminating pre‐drying requirements. However, this approach typically results in poorly developed pore structures and relatively lower surface areas^[^ ^113^ ^]^ compared to pyrolytic methods, creating a trade‐off between functional groups and textural properties. Zhang et al. combined hydrothermal carbonization with multi‐salt activation to fabricate Ni─N─C SACs with varying porosity.^[^ ^114^ ^]^ The process involved hydrothermal carbonization of dry glossy privets in an autoclave containing water and H_3_PO_4_, which were acquired by vacuum filtration, washed, dried, ball‐milled to powder and sieved. The powder was mixed with KHCO_3_, LiCl, and KCl through ball milling followed by pyrolysis at 900 °C. The obtained products were then acid‐etched ultrasonically with agitation, filtered, washed, and dried. The resulting products were dispersed in Ni(NO_3_)_2_.6H_2_O, dissolved in ethanol, sonicated, stirred and centrifuged to obtain powder, which was then ultrasonically mixed with urea in a mass ratio of 1:10 and pyrolyzed at 800 °C to produce Ni─N─C SACs with 0.308 wt.% Ni content, NiN_4_ coordination and SSA of 1029 m^2^ g^−1^. Ma et al. further optimized hydrothermal approaches using microalgae by integrating freeze‐drying and nitrogen‐doping using g‐C_3_N_4_ as both pore‐forming agent and sacrificial template.^[^ ^115^ ^]^ Their systematic temperature study showed the role of pyrolysis temperature conditions, with 900 °C treatments achieving optimal surface areas (556.676 m^2^ g^−1^) and layered structures compared to lower temperatures. The comparison with g‐C_3_N_4_‐free catalysts (356.111 m^2^ g^−1^) demonstrated the templating effect, providing valuable insights for template‐assisted synthesis strategies.

In summary, biomass‐derived porous carbon supports provide a versatile and tunable platform for the development of SACs, with their structural and chemical properties being readily modulated through precursor selection, activation strategies, and synthetic conditions. Effective SAC synthesis relies on achieving strong metal‐support interactions via functional groups, spatial confinement, and heteroatom coordination. Overall, rational design of biomass carbon supports, tailored by engineering approaches, activation, and doping, offers powerful opportunities for synthesizing high‐performance SACs toward catalytic applications.

## 3 Characterization Techniques for Biomass‐Derived SACs

The investigation of the structure and behaviour of SACs depends on the application of a series of advanced characterization techniques. Specifically, X‐Ray absorption spectroscopy (XAS), including both X‐Ray absorption spectroscopy near‐edge structure (XANES) and EXAFS provides insight into the local atomic environment of single metal centers.^[^ ^116^ ^]^ The sensitivity of the technique to coordination geometry and electronic structure makes it valuable for SAC characterization across different metal systems. For instance, XANES analysis can reveal the oxidation states of metal,^[^ ^48^ ^]^ which is important in understanding the catalytic activity, as the oxidation state can influence the binding energies and reaction pathways. EXAFS analysis can also show the coordination environments of metals in SACs. As an example, Song et al. showed that the R‐space profile of Co─N─C SACs exhibited a primary peak at approximately 1.4 Å assigned to Co─N coordination in the first shell and a secondary peak at 2.3 Å corresponding to Co─C coordination in the second shell.^[^ ^48^ ^]^ Quantitative fitting revealed a Co─N coordination number of 3.6, suggesting CoN_4_ and CoN_3_ moieties, indicating that single atoms can occupy multiple coordination sites. More complex coordination environments were observed in other systems. Fe─N─C SACs demonstrated Fe─N coordination peaks at 1.45 Å with a local bonding configuration of FeN_3_S.^[^ ^44^ ^]^ Besides, X‐ray Photoelectron Spectroscopy (XPS) provides surface‐sensitive details complementing the bulk‐sensitive XAFS analysis,^[^ ^71^ ^]^ while Inductively Coupled Plasma (ICP) analysis can give the loading content of single metal atoms.

In addition to spectroscopic techniques, HAADF‐STEM allows for direct visualization of single atoms dispersed on the support and aids in correlating morphology with catalytic performance. The Z‐contrast imaging of the technique allows individual atoms to appear as bright spots against the carbon support.^[^ ^117^ ^]^ For instance, STEM imaging revealed isolated bright spots distributed across the carbon support, with Energy‐dispersive X‐ray spectroscopy imaging confirming uniform distribution of Co, N and O elements in Co─N─C SACs without apparent agglomeration.^[^ ^48^ ^]^

The combination of HAADF‐STEM with electron energy loss spectroscopy (EELS) provides additional chemical information at the atomic scale. For example, in Fe─N─C SACs, EELS analysis detected N and S atoms near Fe centers, corroborating the EXAFS findings of FeN_3_S coordination.^[^ ^44^ ^]^ Meanwhile, High‐resolution (HR) TEM and SEM revealed the characteristics of the support structure.^[^ ^62^ ^]^ X‐ray diffraction (XRD) has also been used as a complementary technique for confirming the absence of metallic nanoparticles and the degree of graphitization,^[^ ^62^, ^65^, ^71^ ^]^ while Raman spectroscopy has been employed to provide insights into carbon structure and defect density.^[^ ^62^, ^71^ ^]^

In sum, the synergistic application of XAS, HAADF‐TEM, ICP, and complementary characterization techniques like XPS, XRD, HRTEM, and Raman is instrumental in revealing the structure of SACs. However, the dynamic behavior of single atoms during catalytic reactions highlights the need for more advanced in situ characterization methods. The coexistence of single atoms with small clusters or nanoparticles, as observed in some systems, requires improved methods for distinguishing and quantifying these different species.

## 4 Synthesis‐Structure Relationships in Biomass‐Derived SACs

The synthesis of high‐performance biomass‐derived SACs relies on understanding the fundamental relationships between preparation methods and the resulting atomic structures. This section systematically examines these critical structure‐directing factors.

### 4.1 SACs via Direct Biomass Conversion

Direct biomass conversion to SACs is widely investigated, mostly by pyrolytic approach, either directly or with dopants, metal precursors, depending on the inherent composition of biomass precursors. Biomass (e.g., Fe‐rich Enteromorpha, Sedum alfredii) with inherent heteroatoms (N/S) and metals can directly form SACs through thermal treatment. An external metal/dopant should be introduced to ensure sufficient metals/heteroatoms for single‐atom loading and coordination structure optimization. The mixing methods include solution‐based or solid‐state mixing to facilitate the interaction of precursors, while the solvent‐free approach is more environmentally friendly. Activation/template‐assisted approaches with reagents like Zn salts, NH_4_Cl, molten salts, hard templates, and soft templates are beneficial for the spatial dispersion, coordination structure engineering and anchoring of atomic metals. Pyrolysis conditions like temperatures and heating atmosphere (N_2_ or N_2_/NH_3_) require precise control for structural optimization. For example, some studies suggest that higher pyrolysis temperatures favor more unsaturated atomic metal sites (M−N_2_C or M−N_3_);^[^ ^70^ ^]^ higher temperatures may generate a metallic phase.^[^ ^55^ ^]^ The type and quantity of heteroatoms (N, S, O) in the biomass precursor further influence coordination geometry and electronic structure. Key challenges remain in precisely controlling these structure‐determining factors. The heterogeneous nature of biomass leads to batch‐to‐batch variability that complicates reproducibility. Standardized protocols for biomass pretreatment and structural analysis will be essential for establishing definitive structure‐property relationships in this promising class of sustainable catalysts.

### 4.2 SACs via Biomass‐Converted Carbon Support‐Mediated Routes

The synthesis of SACs using biomass‐derived carbon supports involves a systematic transformation of raw biomass into engineered porous carbon matrices, followed by precise metal anchoring. This approach enables tailored control over the structural and chemical properties of SACs. Biomass‐derived carbon matrices can be tailored by activation using reagents like KOH and KHCO_3_, generating high SSAs and highly porous structures. The activation reagents, in combination with activation temperature, critically determine the final macro/meso/microporous architecture. Compared with pyrolysis, hydrothermal carbonization retains high O functional groups but yields lower SSAs. The interaction between metal precursors and functionalized carbon supports dictates SAC formation by coordinating metal species with heteroatoms (e.g., N, O, S).

Metal incorporation strategies exhibit equally profound structural effects. Chelation‐assisted loading (e.g., glucose‐metal ion complexes on carbon surface) creates uniform single‐atom distributions by leveraging oxygen‐rich surfaces, achieving loadings up to 12 wt.% without aggregation.^[^ ^95^ ^]^ The choice of nitrogen source during secondary pyrolysis proves crucial for M−N_4_ coordination, which can be further modulated by the carbon support's defect density. These structure‐controlled synthesis pathways enable systematic optimization of SAC properties for target applications while maintaining the sustainable advantages of biomass precursors.

In summary, carbon‐based supports derived from biomass have proven to be effective for loading metal atoms. To achieve optimal performance of SACs, it is important to strategically engineer the carbon structure. This involves optimizing factors such as pore structure, surface area, metal content and metal coordination. Additionally, the synthesis strategy should be specifically tailored to the specific biomass source used as well as the intended application to enhance effectiveness (**Table** **1**).

**Table 1:** A summary of synthesis strategies for biomass‐derived SACs.

SAC	Biomass	Other Reagents	Involved synthesis approach	Application	Metal load [wt.%]	Structure features	SSA [m^2^g^−1^]	Pore volume [cm^3^ g^−1^]	Coordination	Ref.
Fe─N─S─C	N, S‐self‐doped Porphyra	FeCl_3_⋅6H_2_O 2 M HCl	Pyrolysis (900 °C), acid etching	ORR	–	Micropores, crumpled thin sheet‐like morphology	1533.70	1.04	Fe─N_3_/S	[44]
Fe─N─C	*Enteromorpha*	0.5 M H_2_SO_4_	Pyrolysis (900 °C), acid etching	Fenton‐like reaction	0.84	Mixed Fe clusters and single‐atomic Fe	–	–	FeN_2_O_2_	[53]
Fe─N─C	*Auricularia auricular‐judae*	Urea, KOH, 2 M HClO_4_	Pyrolysis (950 °C), acid etching	ORR	0.10	Nanoporous sheet‐like structure	1106.90	–	Fe─N_4_	[57]
Fe─N─C	*Enteromorpha*	NaHCO_3_, glucose, melamine	Pyrolysis (800 °C)	Fenton‐like reaction	1.30	Mesopores, laminate architecture	609.10	–	Fe─N_4_	[109]
Cu─N─C	Zn self‐doped Waste adsorbent	Dicyandiamide	Pyrolysis (900 °C)	Fenton‐like reaction	3.41	Mesoporous	182.80	–	Cu	[118]
Fe─N─C	*Enteromorpha*	KOH, 1 M H_2_SO_4_	Microwave heating, carbonization (400 °C) pyrolysis (800 °C), acid etching	Fenton‐like reaction	–	Microporous, disordered carbon layers	1176.50	–	Fe─N_4_	[93]
Fe─N─C/clusters	Fe‐self doped *Enteromorpha*	1 M of urea	Urea saturation‐freeze drying, pyrolysis (900 °C)	Fenton‐like reaction	–	–	–	–	Fe─N_3_─C	[43]
Fe─N─C	Fe‐self doped *Enteromorpha*	2.5 M or 5.0 M of urea	Urea‐saturation‐freeze drying, pyrolysis (900 °C)	Fenton‐like reaction	1.6	–	36.39	0.0998	Fe─N_4_─C	[56]
Fe─N─C	*Myriophyllum aquaticum*	K_2_FeO_4_, 2 M HCl	Pre‐carbonization, (400 °C) ball milling, pyrolysis (800 °C), acid etching	Fenton‐like reaction	2.40	Thin carbon layer, hierarchical porous structure	2040.00	–	–	[52]
Fe─N─C	Corn silk	ZnCl_2_, Fe (NO_3_)_3_·9H_2_O, and melamine	Pyrolysis, (800 °C), acid etching	ORR/ OER	4.3	Macro/meso/microporous, 3D hollow structure	912.00	–	Fe─N_4_	[73]
Fe─N─C	Chlorella vulgaris	FeCl_3_.6H_2_O	Ball milling, pyrolysis	Fenton‐like reaction			624.88	–	Fe─N_4_	[65]
Fe─N─C	Rice husk	KHCO_3_, H_2_O_2_	Pyrolysis	Fenton‐like reaction		Microporous			Fe─N_5_	[110]
Fe/Mo─N─C	Legume root nodules	ZnCl_2_	Pre‐carbonization (350 °C), pyrolysis (1000 °C)	ORR	–	Disordered carbon layers, micro/mesoporous	1835.00	1.19		[49]
Mn─N─C	*Phytolacca americana*	Urea, 3:1 biomass/ urea. MnCl_2_·4H_2_O	Pre‐carbonization (700 °C), pyrolysis (700 °C)	Fenton‐like reaction	–	Irregular mesoporous structure	56.54	–	Mn−N_4_	[63]
Mn─N─C	*Phytolacca americana*	Mn, N‐self doped	Pre‐carbonization (250 °C), pyrolysis (750 °C), oxidation (165 °C)	Photocatalysis	1.13	Microporous	470.50	–	Mn−N_4_	[50]
Fe─N─C	Pig liver	Fe (NO_3_)_3_, bi‐component NaCl/KCl salt	Ball milling, pyrolysis (800 °C), acid etching	ORR	2.6	Winding carbon sheets with nanopores	803.10	0.94	Fe–N_4_	[42]
Mn─N─C	*Spirulina*	Dicyandiamide, K_2_CO_3_	Dicyandiamide‐saturation, ball milling and pyrolysis, (700 °C)	Fenton‐like reaction	–	Partially collapsed honeycomb structures	–	–	Mn─N_4_	[51]
Fe─N─C	Chitosan	SiO_2_, Fe phenanthroline, and Zn salt	Sol‐gel, freeze drying, pyrolysis	ORR	0.72	Nanowrinkled, mesoporous	609.00	–	FeN_4_	[23]
Fe─N─C	Waste ferns contaminated by iron mines.	Poly‐block polymer (P123), 0.5 M H_2_SO_4_	Sol‐gel, carbonization, (800 °C)	Photocatalysis	–	Graphitized carbon, micropores (1.03 nm) and mesopores (14.97 nm	275.60	–	FeN_4_	[88]
Fe─N─C	Sodium Alginate	Fe precursor, melamine, H_2_SO_4_	Sol‐gel, freeze drying, pyrolysis, acid etching	ORR	–	Layered structure, micropores	1364.00	–	FeN_4_	[90]
Fe─N─C	Lotus root	SiO_2_, FeCl_2_, melamine, HF	Sol‐gel, freeze drying, pyrolysis (950 °C), acid etching	Electrocatalysis	1.30	Mesoporous (10 nm) honeycomb‐like structure	699.80	–	FeN_4_	[41]
Co─N─C	Silica xerogel	Glucosamine hydrochloride, sodium gluconate and CoCl_2_·6H_2_O, TEOS (SiO_2_ precursor) NH_3_, urea, HF	Sol‐gel, freeze drying, carbonization (900 °C), acid etching, 2nd carbonization	ORR	2.38	3D porous distorted multilayer structures, micro/mesoporous (2‐10 nm)	737.00	–		[61]
Co─N─C	Gelatine	SiO_2_, HF	Sol‐gel, freeze drying, pyrolysis (900 °C), acid etching	Electrochemical catalysis	1.20	Microporous defects and nano‐wrinkles, mesopores (5‐10 nm)	827.20	–	60% CoN_4_ and 40% CoN_3_	[48]
Co─N─C	Corncob	Dicyandiamide and Co (NO_3_)_2_⋅6H_2_O	Doping, ball milling, pyrolysis	Fenton‐like reaction	1.79	Stacked layered architecture, mesopores	17.22		CoN_4_	[99]
Co─N─C	Straw	Melamine, ethanol, HCl	Pyrolysis	Fenton‐like reaction	1.25	Mesoporous	839.7	0.799	CoN_4_ (pyrrolic N)	[119]
Co─N─B─P─C	Ganoderma lucidum spores	Phytic acid, urea, boric acid	Hydrothermal, pyrolysis	OER	–	Mesoporous	48.5469			[120]
Fe─N─C	Furfural residues	Phenanthroline‐N, KOH, ethanol, melamine, 1 M H_2_SO_4_	Pre‐treatment‐pyrolysis, (600 °C), ball milling, pyrolysis, (800 °C), wetness impregnation, ball milling, pyrolysis (700 °C), acid etching	Fenton‐like reaction	–	Microporous	507.76	0.27	Fe─N_2_─O_2_	[104]
FePt/N–C	Porphyra	Urea, HCl Porphyra‐Fe‐self‐doped	Impregnation, freeze drying, pyrolysis (950 °C), acid etching	HER, ORR	Fe (0.166) Pt (2.29)	Micro‐ and mesopores	1009.00	–	Fe−N_4_ and Pt−N_4_	[121]
Fe─N─C	Lotus seedpod	Spend acid (FeCl_3_, HCl)	Impregnation, freeze drying, pyrolysis (900 °C)	ORR	∼0.55	Micro/mesopores heteroarchitectural microstructure with roughness	2465.60	1.43	–	[60]
Fe─N─C	Porphyra	KOH, Dimethylformamide (DMF), HF	Pre‐carbonization (900 °C), impregnation, pyrolysis (800 °C)	ORR	2.30	Micropores and mesopores	1449.10	0.77	–	[94]
Fe─N─C	Microalgae		Hydrothermal pre‐treatment (180 °C), freeze‐drying, pyrolysis, (900 °C)	ORR	1.98	Layered, hierarchical porous structure	556.68	0.33	–	[115]
Fe─N─C	Waste pig blood	Zn (NO_3_)_2_⋅6H_2_O Zn salt, 3 M HCl	Hydrothermal pre‐treatment (200 °C), acid‐treatment, incubation (950 °C) in N_2_‐NH_3_	ORR	1.30	2D porous sheet‐nanostructure and curved graphene layers	1260.00	2.94	–	[46]
Fe–O–C	Cellulose	Metal nitrate solutions, 2 mol L^‒1^ H_2_SO_4_	Hydrothermal treatment (240 °C) pyrolysis (800 °C), acid ‐treatment	Photocatalysis	0.91	Micropores (0.5 and 2 nm) and mesopores between (2.5‐4.5 nm)	388.00	–	FeO_3_C	[47]
Fe–N–O–C	Cotton stalk	Melamine	Ball milling, pyrolysis	Fenton‐like reaction	1.3	Lamellar wrinkled			FeN_3_OC	[66]
Co─N─C	Chitosan	F127 (surfactant), HCl, ethanol, 4‐ aminophenol, HMT, acetic acid and Co salt	Hydrothermal treatment, freeze‐drying, pyrolysis, acid etching	ORR	1.88	3D cross‐linked coral structure, irregular sheet wedge‐shaped or fissure‐shaped pore structure	371.75	0.504	–	[89]
Co─N─C	Wastepaper	Chloroacetic acid, NaOH, PEI‐ N precursor, TCCP‐Co, 1 M H_2_SO_4_	Hydrothermal treatment, sonication, (65 °C) 4 h, oven‐drying, calcination (550 °C, 700 °C), acid etching	Fenton‐like reaction	–	Sea cucumber‐like mesoporous structure	535.20	–	Co─N_3_	[122]
Fe─N─C	Wood	FeCl_3_, NH_3_	Wet impregnation, carbonization, acid etching, pyrolysis	ORR/OER	∼0.80	Layered structure, rough surface, micropores and mesopores	1183.23	0.86	–	[59]
Co─N─S─C	Spent coffee grounds	Cobalt chloride hexahydrate (CoCl_2_·6H_2_O)	Wetness impregnation and drying, carbonization (500 °C)	Fenton‐like reaction		–	293.99	–	Co─N_3_S_1_	[24]
Co─N─C	Chitosan	NH_4_Cl, Co (Ac)_2._ 4H_2_O and acetic acid	Wetness impregnation, freeze‐drying, carbonization (850 °C)	Electrocatalysis	1.27	3D ultrathin hierarchically macro/meso/microporous texture,	1977.90	2.99 m^3^ g^‒1^	CoN_4_	[75]
Co─N─C	Chitosan	ZnCl_2_ salt	Wet impregnation, drying, carbonization (900 °C), acid etching,	Oxidation of hydrocarbons	0.10	Porous belt‐like nanostructure	2513	2.20	CoN_4_	[72]
Co─N─C	Cocoon silk	ZnCl_2_ salt 1 M HCl CoCl_2_	Wet impregnation, drying, pyrolysis (900 °C), acid etching	Oxidation of benzene	0.60	Sheet‐like 2D structure with uniform morphology and mesopores	2105.00	1.70	CoN_4_	[27]
Co─N─C	Lignin	Co (NO_3_)_2_.6H_2_O and Zn (NO_3_)_2_.6H_2_O, dicyanamide	Wet impregnation, drying, carbonization, (500 °C, 1000 °C)	Oxidative esterification	1.54	Disordered carbon support	–	–	CoN_3_	[26]
Co─N─C	Lignin	Cobalt (II) acetate and zinc nitrate, dicyanamide	Wet impregnation, drying, carbonization, (500 °C, (1000 °C)	Fenton‐like reaction	2.35	–	–	–	CoN_3_	[25]
Co─N─C	Lignin	Melamine‐N, ZnCl_2_	Hydrolysis, freeze drying, pyrolysis	H_2_ production from methanol	1.00	Laminar architecture and fluffy structure	–	–	CoN_4_	[40]
Co─N─B─C	Starch	Dicyandiamide, S, boric acid, SiO_2_	Wet impregnation, freeze‐drying, pyrolysis (900 °C) acid etching (HF, 3 M), H_2_‐activation pyrolysis, (H_2_/Ar, 600 °C)	OSI, ODH, THG	1.90	3D and porous structure, mesopores	683.30	1.48	CoN_3_B_1_	[85]
Co─N─C	*Chlorella*	NaCl/KCl	Impregnation, freeze drying, pyrolysis (800 °C)	ORR	3.10	Average pore size 6.3 nm, crumpled sheet‐like morphology	2907.00	6.30	Co─N_4_	[83]
Zn─N─C	Lignin	Dicyandiamide	Wetness impregnation, drying, carbonization (1000 °C)	Cleavage of C─N bonds	0.67	2D thin layer porous structure	584.00	0.96	Zn─N_4_	[71]
Mn─N─C	Semen sterculia lychnopherae	EDTA, melamine (g‐C_3_N_4_)	Impregnation, freeze drying, pyrolysis (500 °C, 800 °C)	Ozonation	7.80	Yarn‐like structure with an ultrathin 2D graphitic carbon layer, with mesopores	123.13	0.64	Mn−N_4_	[62]
Cu─N─C	Cellulose	1 M H_2_SO_4_, phenanthroline	Wet impregnation, drying, pyrolysis (500 °C)	Fenton‐like reaction	4.08	Irregular and roughly Wrinkled surface with a porous bulk structure, Mesoporous structure	–	–	Cu─N_4_	[58]
Ni─N─C	Lignin	Dicyandiamide	Wet impregnation, oven drying, carbonization (1000 °C)	CO_2_RR	0.43	–	–	–	Ni─N_2_C	[70]
Ni─N─C	Privets	KHCO_3_ and LiCl/KCl	Hydrothermal carbonization, pyrolysis (800 °C)	CO_2_RR	0.308	Hierarchical pores	1029	–	NiN_4_	[114]
Co─N─C	Lignin	Co(NO_3_)_2_⋅6H_2_O, Zn(NO_3_)_2_⋅6H_2_O, Dicyandiamide, H_2_SO_4_	Carbonization (550 °C, 1000 °C)	Fenton‐like reaction	0.24 at.%	Curly sheet	305.3301	–	Co─N_3_	[69]
Co─N─C	Walnut shells	Co(NO_3_)_2_, Dicyandiamide, H_2_SO_4_	Carbonization (550 °C, 1000 °C)	Fenton‐like reaction	–	–	41.9262	–	Co─oxo	[98]
Co─N─C	Rice straw	o‐phenanthroline, ethanol	Pyrolysis (600 °C)	Fenton‐like reaction	0.57 at.%	Lumpy structure		–		[97]
Co─N─C	Chitosan	acetic acid, H_2_SO_4_	Pyrolysis (600 °C), NH_3_/Ar	Fenton‐like reaction	2.9	–	–	–	Co─N_3_	[55]
Co/Cu─N─C	Cow dung	Dicyandiamide, H_2_SO_4_	Carbonization (550 °C, 950 °C)	Fenton‐like reaction	Cu 1.71, Co 0.47	Macroporous and mesoporous structure, lamellar structure	Co─N─C 95.4, Cu─N─C 89.2, Co/Cu─N─C 103.5	–	Co─N_3_ and CuN_3_O_2_	[123]
Fe─N─C	Lignin (vanillin)	HCl, KOH	Pyrolysis (800 °C)	Fenton‐like reaction	0.65	Honeycomb‐like porous structure	2612.7	–	Fe─N_4_ (pyrrolic N)	[103]
Fe─N─C	Shrimp shells	Melamine‐cyanuric acid	Pyrolysis (550 °C)	Fenton‐like reaction	0.24 at.%	Block‐like structure	27.8	–	Fe─N_4_ (pyridinic N)	[124]
Fe─N─C	Chestnut shells	KOH		Fenton‐like reaction	6.34		1470.25	–	Fe(III) ─N_4_	[102]
Cu─N─C	Cellulose	Dicyandiamide, HCl	Pyrolysis (550 °C, 900 °C)	Fenton‐like reaction		Flat‐2D carbon layers	182.8		Cu─N_4_ (pyridine N)	[125]
Cu─N─C	Blue‐green algae	Melamine, NaCl/KCl, cyanuric acid	Pyrolysis (800 °C)	Fenton‐like reaction		Hierarchical pore structure	565.1			[84]
Fe─N─C	Rice husk and lignin	Dicyandiamide, H_2_SO_4_, Zn(NO_3_)_2_⋅6H_2_O		Fenton‐like reaction		Curled lamellar structure	156.98	0.32	Fe─N_4_	[96]
Fe─N─C	Hyperaccumulator residues		Pyrolysis (800 °C)	Fenton‐like reaction	1.41	Smooth‐edged sheet structure	25.52		Fe─N_3_─O_1_	[54]
Fe─N─C	Duckweed	KOH, HCl, citric acid, ethylene glycol	Pyrolysis (900 °C)	Fenton‐like reaction	Fe 1.16, Ce 0.78		1260			[126]

## 5 Applications of Biomass‐Derived SACs in Fenton‐Like Reactions

Fenton‐like reactions with PMS, peroxydisulphate (PDS), or H_2_O_2_ as the oxidants provide a sustainable method for removing recalcitrant organic micropollutants from water.^[^ ^127^ ^]^ However, the degradation of micropollutants by PMS/PDS/H_2_O_2_ alone is slow.^[^ ^128^ ^]^ These oxidants can be activated by ultraviolet light,^[^ ^129^ ^]^ or other external energy sources such as electrical energy, ultrasound,^[^ ^130^ ^]^ alkali,^[^ ^131^ ^]^ carbon‐based materials,^[^ ^130^, ^132^ ^]^ and transition metal‐based catalysts.^[^ ^133^, ^134^ ^]^ In recent years, biomass‐derived SACs, are gaining attention due to their environmental benefits, cost‐effectiveness, and high catalytic efficiency with atomic metal loading. They have been demonstrated as efficient catalysts in PMS, PDS, or H_2_O_2_ activation to generate ROS,^[^ ^128^, ^135^ ^]^ or electron transfer pathway (ETP) for degrading micropollutants. This section explores the correlation between active sites on biomass‐derived SACs and the corresponding degradation mechanisms in different Fenton‐like systems, aiming to provide some guidelines for future developments of SACs in this area. **Table** **2** summarizes the research works on the activation of PMS/PDS/H_2_O_2_ with biomass‐derived SACs for degrading various organic micropollutants under different reaction conditions.

**Table 2:** Summary of biomass‐derived SACs and the respective dominant reactive species generated via PDS/PMS activation.

SACs	Oxidants	PMS/PDS	Catalyst	Pollutant	Active sites	Reaction pathway	TOF	K_obs_ [min^−1^]	Removal efficiency	Cycles	Ref.
Co─N─C	PDS	0.5 g L^−1^	0.2 g L^−1^	20 mg L^−1^	Co─N_3_	^•^OH, SO_4_^•−^ and ETP		0.16	93%, 1 min	5 cycles (>95%) 5	[122]
Co─N─S─C	PMS	2 mM	1.0 g L^−1^	2 mg L^−1^	Co−N_3_S_1_	SO_4_^•−^, ^•^OH (major) ^1^O_2_ (<10%) (minor)			PCB28 (90%, 128 min), DEP (90%, 60 min) and BPA (100%, 30 min)	PCB28 ‐second cycle (90%), third cycle (60%)	[24]
Co─N─C	PMS	0.5 mM	0.05 g L^−1^	10 mg L^−1^	CoN_3_(II)	ETP		0.241	100% NPX, PCM, CIP, and BPA, 40–60% of CP and MNZ (40‐60%) 90 min	4 cycles	[25]
Co─N─C	PMS	0.6 mM	0.4 g L^−1^	10 mg L^−1^	Co─N_4_	^1^O_2_		0.444	98.5%, 10 min	5 cycles (88.4%)	[99]
Co─N─C	PDS	2 mM	0.05 g L^−1^	10 mg L^−1^	Co─N_4_ (Pyrrolic N)	^1^O_2_ and ETP		0.410	(TCH, 10 mg L^−1^) 100% in 6 min. TCH (25 mg L^−1^) 96.3% in 10 min at 4 °C.		[119]
Co─N─C	PMS	0.8 mM	0.1 g L^−1^	45 µM	Co─N_3_	High‐valent Co species		1.1	100%, 5 min	4^th^ cycle (70%)	[55]
Co─N─C	PMS	1 mM	0.1 g L^−1^	10 mg L^−1^	Co─N_3_	ETP			97.5% within 30 min	4 cycles (95% after regeneration)	[69]
Co─N─C	PMS	0.5 mM	100 mg L^−1^	50 mM	Co─N_4_	High‐valent Co‐oxo species			100%, 60 min	4 cycles	[98]
Co─N─C	PMS	0.4 mM	0.1 g L^−1^	39.48 µM	Octahedral Co	^1^O_2_ and SO_4_^•−^ and ETP	1.267		99.8%, 60 min	5 cycles (96.9%)	[97]
Co/Cu─N─C	PMS	1.2 mM	0.02 g L^−1^	15 mg L^−1^	Co─N_3_/ CuN_3_O_2_	Co/Cu─N─C SO_4_^•−^, ^•^OH, Co─N─C SO_4_^•−^, Cu─N─C ^1^O_2_				4 cycles (>85.1%)	[123]
Fe─N─C	PMS	0.5 mM	0.05 g L^−1^	100 mg L^−1^	Single Fe atoms	^1^O_2_			96.1%, 60 min	5 cycles (76%)	[103]
Fe─N─C	PMS	0.5 g L^−1^	0.2 g L^−1^	10 mg L^−1^	Fe─N_4_ (pyridinic)	ETP		0.53	94.5%, 5 min	─	[124]
Fe─N─C	PMS	1 mM L^‒1^	0.1 g L^−1^	10 mg L^−1^	Fe─N_4_	High‐valent iron‐oxygen species			100%	─	[102]
Fe─N─C	PMS	0.15 mM	0.1 g L^−1^	20 mg L^−1^	Fe─N_4_	ETP			91%	4 cycles (75%)	[96]
Fe─N─C	PMS	1 mM	0.2 g L^−1^	10 mg L^−1^	FeN_3_O_1_	ETP	0.79		100%, 30 min	4 cycles (100%)	[54]
Fe/Ce─N─C	PMS	0.4 g L^−1^	0.1 g L^−1^	40 mg L^−1^	Single atomic Fe/Ce sites	^1^O_2_, SO_4_^•−^, ^•^OH		Ce─N─C = 0.133; Fe─N─C = 0.1204	100%, 30 min	4 cycles (above 80%)	[126]
Fe─N─C	Photocatalysis/PMS	8 mg	10 mg	20 mg L^−1^	Fe─N_4_	^1^O_2_ and O_2_^•−^ (major), SO_4_^•−^, ^•^OH (minor)		0.031	NOR (87.2%), LEV (91.4%), CIP (88.5%), ENR (100%), LOM (99.4%), FLU (90.3%) in 1 h	4 cycles	[88]
Fe─N─C	PMS	0.5 mM	0.05 g L^−1^	10 mg L^−1^	FeN_2_O_2_	^1^O_2_	1.72		TOF (1.72 min^− 1^) 96.8%, 40 min	Several cycles	[104]
Fe─N─C	PDS	0.4 mM	40 mg L^−1^	20 µM	Fe‐pyridinic N_4_	ETP		0.153	97.1%, 20 min	70% after 20 h	[109]
Fe─N─C	PMS	0.5 mM	0.1 g L^−1^	10 mg L^−1^	FeN_4_	^1^O_2_ and ETP (major) SO_4_^•−^, ^•^OH and O_2_^•−^ (minor)		0.081	100%, 60 min	5 cycles (63.37%)	[93]
Fe─N─C/clusters	PMS	0.5 mM	0.1 g L^−1^	10 mg L^−1^	Fe–N_3_–C	ETP (major), SO_4_^•−^ and ^•^OH (minor)		0.282	100%, 20 min	4 cycles (95%)	[43]
Fe─N─C	PMS	0.5 mM	0.1 g L^−1^	10 mg L^−1^	Fe_2_N_2_O_2_	ETP			PCM 100%, 45 min; CIP, sulfamethazine, and BPA, 100% within 90 min; CP 20%, 90 min	PCM 4th cycle (90%) and 5th cycle (80%) 180 min	[53]
Fe─N─C	PMS	0.5 g L^−1^	0.05 g L^−1^	20 mg L^−1^	–	ETP		0.123	100%, 6 min, 100%, 60 min for SD and HBA	(Phenol) 1^st^ cycle (100%), 2^nd^ cycle (64%), and 3^rd^ cycle (5%), 6 min	[52]
Fe─N─C	PMS	1.0 mM	0.1 g L^−1^	10 mg L^−1^	Fe–N_4_–C	ETP Fe (IV) and ^1^O_2_ (minor)		0.407	90.46%		[56]
Fe─N─C	H_2_O_2_	0.3 mM	50 mg L^−1^	20 µM	Fe–N_4_	^•^OH, and ^1^O_2_			100%, 30 min		[65]
Fe─N─C	PMS	75 mg L^−1^	80 mg L^−1^	20 mg L^−1^	Fe–N_5_	ETP			100%, 6 min	4 cycles (>98%)	[110]
Fe─N─C	PMS	0.5 g L^−1^	0.1 g L^−1^	20 mg L^−1^	Fe–N_3_O–C	^1^O_2_			99%, 5 min		[66]
Mn─N─C	PMS	2 mM	1.0 g L^−1^	25 mg L^−1^	Mn−N_4_	^•^OH, SO_4_^•−^ and ^1^O_2_			90%, 30 min	Second cycle (58.86); third cycle (61.15%), 30 min	[63]
Mn─N─C	PMS	1 mM	0.2 g L^−1^	10 mg L^−1^	MnN_4_	O_2_^•−^ and ^1^O_2_ (major), SO_4_^•−^, ^•^OH (minor)		0.31	98% E NR and 95% CIP, 10 min; 100% BPA, 8 min, 15% BA 10 min	ENR 3 cycles (80%)	[51]
Cu─N─C	PMS	0.4 g L^−1^	0.1 g L^−1^	20 mg L^−1^	Cu–N_4_	ETP			100%, 30 min	5 cycles (>97%)	[118]
Cu─N─C	PMS	0.05 mM	0.2 g L^−1^	10 mg L^−1^	Cu–N_4_	^•^OH and ^1^O_2_ (major), SO_4_^•−^ (minor)			96.47%, 60 min	3 cycles (63.98%) (pH 5–9)	[58]
Cu─N─C	PDS	0.2 g L^−1^	0.1 g L^−1^	20 mg L^−1^	Cu–N_4_	ETP			100%, 30 min	–	[125]
Cu─N─C	PDS	1 mM	0.05 g L^−1^	20 mg L^−1^		O_2_^•−^, ^1^O_2_		0.025	87%, 90 min	4 cycles (71.8%)	[84]

### 5.1 PMS Activation

PMS is an oxidant with an asymmetrical structure.^[^ ^136^ ^]^ Recently, M─N─C SACS (M = Fe, Co, Mn, and Cu) have been used to activate PMS to degrade different micropollutants. The degradation pathway can be classified into nonradicals and free radicals. The non‐radical pathways include ETP, singlet oxygen (^1^O_2_), high‐valent metal species, and surface active complex.^[^ ^137^ ^]^ Free radicals include hydroxyls (^•^OH), sulphates (SO_4_^•−^), and superoxide anion (O_2_^•–^).

#### 5.1.1 Biomass‐Derived Fe‐Based SACs for PMS Activation

Fe‐based catalysts have emerged as fundamental catalysts for PMS activation, showcasing distinct active sites on their surfaces. Their performance has been shown to depend largely on their configuration.^[^ ^66^ ^]^

Fe─N_4_ coordination is among the most widely studied configurations in SACs, due to its stable geometry.^[^ ^138^ ^]^ Internal single‐atom Fe can enhance the adsorption properties of the catalyst and optimize electron transfer,^[^ ^139^ ^]^ facilitating improved catalytic performance. Tian et al. showed that Fe─N─C SACs synthesized via microwave heating of Fe‐rich *Enteromorpha* achieved complete degradation of chloroquine phosphate (CQP) in 60 min.^[^ ^93^ ^]^ The microwave‐assisted synthesis created a hierarchical porous structure with abundant defects and high Fe^2+^ availability, enhancing PMS activation for the generation of ^1^O_2_ through an electron transfer‐mediated process. The effectiveness of the Fe─N_4_ active sites was improved by structural defects generated through microwave “hotspots”. Similarly, in another study, Fe─N_4_ configuration acted as the major reactive site for norfloxacin (NOR) degradation via a photocatalytic process.^[^ ^88^ ^]^ Results showed that PMS preferentially bonds to Fe─N_4_ through an intermediate interaction, enhancing PMS activation through the generation of ^1^O_2_, O_2_^•−^, ^•^OH, and SO_4_^•−^. Of the four ROS, ^1^O_2_ and O_2_^•−^ were the main active species responsible for degrading NOR. The Fe─N─C SAC/PMS system could degrade 87.2% of NOR within 1 h under light irradiation. The catalytic performance from the synthesized Fe─N─C SACs was primarily attributed to the synergistic effects of the Fe─N_4_ coordination structure, the hierarchical porous structure, and partially graphitized porous carbon. These features facilitated visible light adsorption and charge carrier separation. The catalyst showed stability over four consecutive runs and demonstrated versatility in PMS activation for the photocatalytic degradation of five other types of quinolone antibiotics (ciprofloxacin (CIP) (88.5%), enrofloxacin (ENR) (100%), levofloxacin (LEV) (91.4%), lomefloxacin (LOM) (99.4%) and flumequine (FLU) (90.3%)). Another study explored the role of pore size distribution in 3D porous materials to enhance the density of Fe─N_4_ active sites in Fe─N─C SACs.^[^ ^102^ ^]^ The synthesized Fe─N─C SACs activated 1 mM PMS for complete oxytetracycline (OTC) degradation via a non‐radical degradation mechanism involving high‐valent iron–oxygen intermediates. Between the high‐valent species formed during the reaction, Fe (IV)─OH, rather than Fe (IV)═O was identified as the dominant active species responsible for OTC degradation. DFT calculations and molecular dynamics simulations revealed a direct correlation between the number of Fe─N_4_ active sites and catalytic performance, emphasizing the importance of structural optimization. Further, the catalyst demonstrated stability, maintaining high catalytic efficiency over ten cycles with Fe leaching below 0.06 mg L^−1^. Fang et al. extended the use of Fe─N_4_ SACs by hybridizing them with biochar, creating a built‐in electric field at the interface between biochar and Fe atom sites, optimizing electron transfer.^[^ ^96^ ^]^ When applied to activate 0.15 mM PMS for tetracycline (TC) degradation, the hybrid system achieved a degradation efficiency of 91% in 90 min. The Fe─N_4_ sites and the electron‐rich C═O groups on biochar synergistically served as the major active sites, facilitating the generation of SO_4_^•−^, ^1^O_2_, and high valent Fe species. Even after four cycles, the catalyst could still achieve 75% degradation efficiency for TC, highlighting its stability. Further, the catalyst exhibited stability across a wide pH range (5–11) with minimal leaching except under extremely acidic conditions, attributed to strong electron interactions within the composite structure.

**Figure 6:**
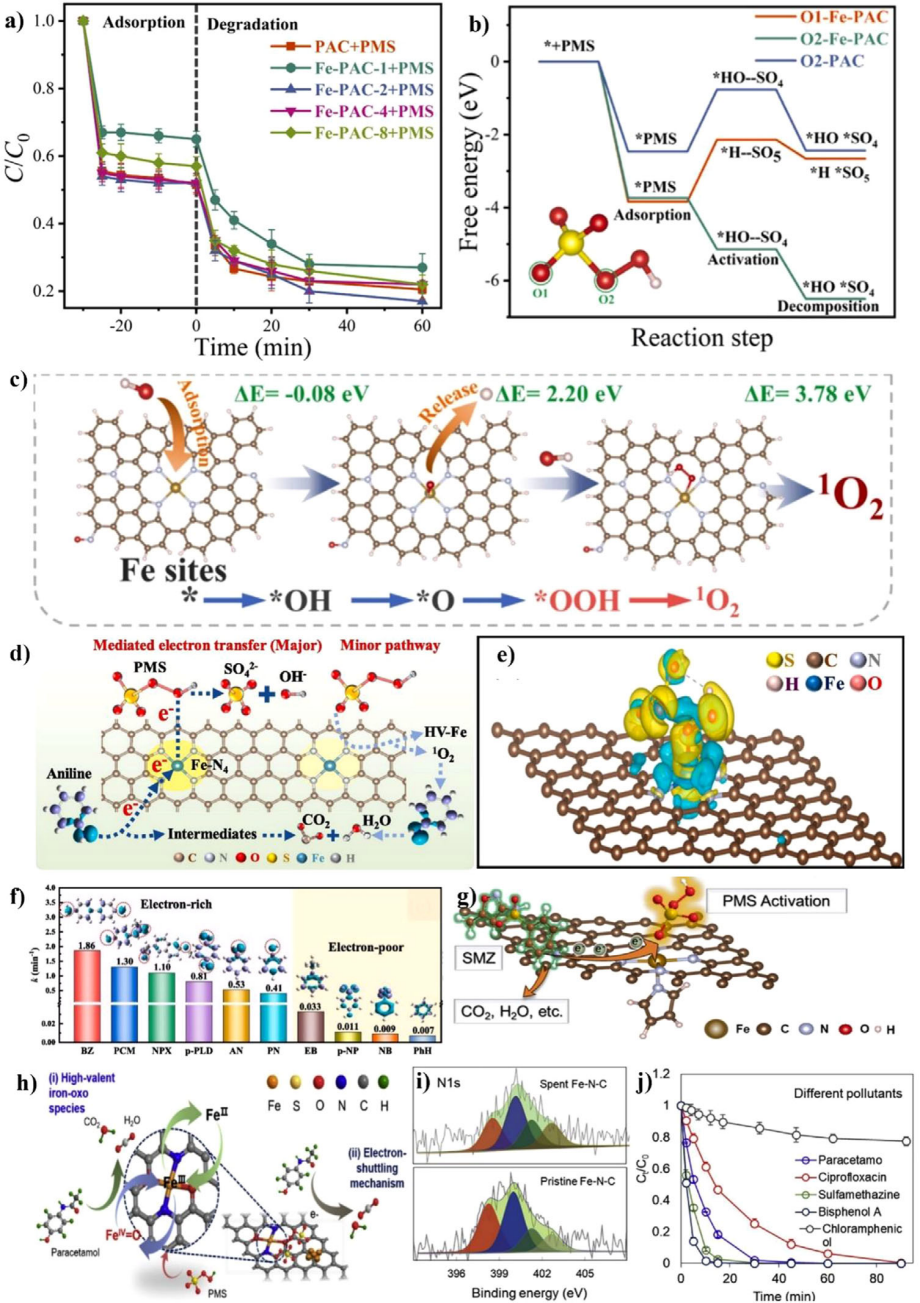
Mechanisms of biomass‐derived Fe‐based SACs for the activation of PMS and the degradation of various organic micropollutants. (a) Schematic illustration of the removal capacity of SMT on different catalysts, (b) Proposed mechanism of ^1^O_2_ generation for the activation of PMS on Fe─N─C SACs by DFT methods and (c) the pathway from *OH to ^1^O_2_ on Fe sites in Fe─N─C SACs. Reproduced with permission.^[^ ^103^ ^]^ Copyright 2023, Elsevier. (d) Catalytic degradation mechanism in Fe─N─C SAC /PMS system. (e) Charge density difference after optimized PMS configuration adsorbing on Fe─N─C SACs (green and yellow represent electron depletion and electron accumulation with the isosurface value of 0.001 e/Bohr,^3^ respectively). (f) Schematic illustrating reaction kinetic constants of different micropollutants. Reproduced with permission.^[^ ^124^ ^]^ Copyright 2024, Elsevier. (g) Scheme showing the catalyst‐mediated electron‐transfer path. Reproduced with permission.^[^ ^110^ ^]^ Copyright 2024, Elsevier. (h) Mechanism scheme for the degradation of PCM via ETP and high‐valent iron‐oxo species. (i) XPS N 1s of Fe─N─C SACs before and after use. (j) Catalytic degradation of different micropollutants in *Enteromorpha*‐derived Fe─N─C SAC/PMS system. Reproduced with permission.^[^ ^53^ ^]^ Copyright 2021, Elsevier.

These studies demonstrate the significance of Fe─N_4_ coordination in enhancing the performance of biomass‐derived SACs by tailoring the coordination environment, pore structure, and hybridization with other materials for enhanced electron transfer, ROS generation, and micropollutant degradation efficiency. Specific N environments within the general Fe─N_4_ coordination systems, such as pyridinic N and pyrrolic N, significantly influence catalytic performance. Liu et al. demonstrated the role of pyrrolic N in enhancing the performance of Fe─N_4_─C SACs for the activation of 0.5 mM PMS for sulfamethazine (SMT) removal.^[^ ^103^ ^]^ The synergistic effects of adsorption and degradation were assigned to the large SSA (2612 m^2^g^‒1^) of the catalyst and single Fe atoms coordinated with pyrrolic N. This configuration facilitated the generation of ^1^O_2_, identified as the dominant ROS. Under optimal conditions, with an Fe loading content of 2 mmol, the Fe─N─C SAC/PMS system demonstrated a removal efficiency of 96.1% in 60 min (**Figure** **6**a). However, excess Fe content led to agglomeration, reducing catalytic performance. Both experimental results and DFT calculations highlighted the mechanism by which Fe─N_4_ sites (pyrrolic N) catalyzed PMS activation. The proposed mechanism of the generation of ^1^O_2_ based on DFT methods shows that Fe sites in Fe─N─C SACs favour adsorption at the O2 site of PMS, leading to the formation of OH* and SO_4_* (Figure 6b). The OH* then transforms into ^1^O_2_ via the following pathway: PMS→OH*→O*→OOH*→^1^O_2_ (Figure 6c). The findings emphasize the importance of maintaining an optimal Fe loading and pyrrolic N content for optimum degradation efficiency.

In comparison, Fe─N_4_ sites coordinated with pyridinic N exhibit distinct catalytic properties. Zhou et al. investigated pyridinic Fe─N_4_ sites in Fe─N─C SACs for aniline (AN) degradation, achieving a 94.5% removal efficiency within 5 min when oxidized by 0.5 g L^‒1^ PMS.^[^ ^124^ ^]^ The electron‐rich pyridinic Fe─N_4_ sites facilitated strong PMS adsorption, forming a metastable complex (Fe─N─C SACs/PMS*). The complex played an important role in mediating electron transfer from AN to PMS, creating an efficient electron shuttle pathway (Figure 6d). Charge density analysis revealed significant electron accumulation (Figure 6e) at the pyridinic Fe─N_4_ sites, enhancing their redox capability compared to graphene. While ETP was dominant, contributing 77.1% of AN degradation, ^1^O_2_ and high‐valent Fe species accounted for the remaining efficiency. Furthermore, degradation efficiency was demonstrated to depend on the energy gap between the Highest Occupied Molecular Orbital (HOMO) of the micropollutant and the Lowest Unoccupied Molecular Orbital (LUMO) of the catalyst (Figure 6f), indicating a relationship between electronic structure and catalytic performance.

The addition of C as a secondary coordination element in Fe─N_4_─C configurations enhances charge transfer and stability. For example, Yin et al. synthesized Fe─N─C SACs with single‐atom Fe in Fe─N_4_─C coordination and demonstrated their catalytic performance in activating 1 mM PMS for naproxen (NPX) degradation via an electron transfer pathway, with minimal contributions from ^1^O_2_ and Fe (IV).^[^ ^56^ ^]^ The use of urea‐treated *Enteromorpha* biomass enriched the nitrogen content, promoting Fe─N_4_─C site formation and minimizing Fe‐Fe clustering during pyrolysis. The catalyst maintained stability across a range of pH conditions (3.0‐6.0) and in diverse water matrices (tap water, natural water, and seawater). Immobilization onto ceramic membranes further increased stability, achieving complete NPX degradation with a relatively lower PMS dosage (0.81 mM). Fe─N─C SACs showed minimal Fe leaching (below 5 µg L^−1^) with degradation efficiency exceeding 95% of its catalytic activity. The Fe─N_4_─C configuration further demonstrates the importance of structural optimization in biomass‐derived SACs.

In an asymmetric Fe─N_3_ site, Fe coordination changes the Fe oxidation state and charge transfer efficiency of SACs,^[^ ^140^ ^]^ leading to an enhanced catalytic performance in SACs.^[^ ^141^ ^]^ Li et al. synthesized Fe─N─C SACs with Fe─N_3_─O_1_ coordination to facilitate 1 mM PMS activation for the selective oxidation of electron‐rich phenolic such as bisphenol A (BPA).^[^ ^54^ ^]^ The Fe─N─C SACs were derived from *Sedum alfredii*, a heavy metal hyperaccumulator sampled from a Zn‐contaminated site and naturally enriched with Fe. This bioaccumulation enabled self‐dispersion of Fe atoms during pyrolysis, forming Fe─N_3_─O_1_ coordination. The degradation was facilitated predominantly via a non‐radical pathway driven by potential differences, with a strong correlation between ionization potential and degradation kinetics. DFT calculations showed that the Fe─N_3_─O_1_ coordination structure acted as the key active site, optimizing the electronic properties by narrowing the energy gap and facilitating electron transfer between BPA and the Fe─N─C SACs/PMS* complex. The catalyst achieved 100% BPA removal within 30 min, outperforming commercial materials such as CNTs, Co_3_O_4_, and reduced graphene oxide. Additionally, the system demonstrated reusability and stability, maintaining complete BPA removal across four cycles. The Fe─N_3_─O_1_ coordination facilitated the selective oxidation of electron‐rich micropollutants such as BPA and 2‐methoxyphenol, with significantly higher reaction rates than electron‐deficient phenol derivatives such as nitrophenol and benzoic acid.

Meanwhile, in some cases, the Fe─N_3_ site can coordinate with C, resulting in Fe─N_3_─C coordination, which shows favourable energetics.^[^ ^142^ ^]^ Zhang et al. utilized Fe─N─C SACs with Fe─N_3_─C active sites to degrade NPX through ETP.^[^ ^43^ ^]^ The Fe─N_3_─C structure provided favorable energetics for adsorption and electron transfer, facilitating 100% NPX degradation within 20 min by activating PMS. The system exhibited a rate constant (k) of 0.282 min^−1^ and maintained 95% of its catalytic capacity over four cycles. When the catalyst was immobilized onto a ceramic membrane, it sustained continuous NPX degradation over 5 L of solution, showcasing both scalability and stability. Comparative studies revealed that catalysts synthesized with urea showed better Fe atom dispersion and higher performance than those prepared without urea, highlighting the role of synthesis methods in optimizing Fe─N_3_─C coordination.

Researchers have sought to improve catalytic performance further by incorporating dual heteroatom doping. This approach leverages the synergy between different functional groups to optimize the coordination environment. For example, Xue et al. synthesized Fe–N–O–C SACs using oxygen‐rich cotton stalk biomass, promoting the formation of a Fe–N_3_–O–C coordination structure.^[^ ^66^ ^]^ This configuration enabled selective PMS activation for acid orange 7 (AO7) via a ^1^O_2_‐mediated non‐radical pathway with negligible contributions from other ROS such as ^•^OH and SO_4_^•−^. The high oxygen content of the precursor enhanced Fe atom dispersion and active site stability, as confirmed by the absence of iron oxide phases.

High‐coordination sites such as FeN_5_ configurations represent another important advancement in the design of biomass‐derived SACs. Du et al. synthesized Fe─N─C SACs with FeN_5_ coordination sites by exploiting rice husk‐derived biochar enriched with micropores, oxygen functional groups and carbon vacancies introduced via KHCO_3_ activation and H_2_O_2_ oxidation.^[^ ^110^ ^]^ These structural features enhanced Fe^3+^ binding and facilitated nitrogen doping. DFT calculations showed that the FeN_5_ configuration provided a more favorable global energy compared to the FeN_4_ configuration, facilitating rapid electron transfer between PMS and sulfamethoxazole (Figure 6g) and resulting in complete degradation of sulfamethoxazole in 6 min. Comparative studies showed that these Fe─N─C SACs outperformed both functionalized and unmodified porous biochar, highlighting the synergy between the tailored porous structure, the Fe center, and nitrogen coordination in driving high catalytic efficiency. Additionally, the catalyst demonstrated robust reusability with over 98% removal efficiency retained after four cycles.

Researchers have also investigated FeN_2_O_2_ coordination sites as effective active centers for biomass‐derived SACs. Besides acting directly as catalytic sites, single metal sites can generate high‐valence metal‐oxo species for further PMS activation.^[^ ^143^ ^]^ Peng et al. synthesized Fe─N─C SACs with FeN_2_O_2_ sites for activating 0.5 mM PMS during paracetamol (PCM) degradation.^[^ ^53^ ^]^ *Enteromorpha*, an Fe‐ and N‐containing marine biomass, was used as the precursor. Despite its low nitrogen content, it enabled the formation of FeN_2_O_2_ coordination sites through high‐temperature pyrolysis. Single Fe atoms were the primary active sites responsible for the formation of high‐valent iron‐oxo species (Fe^IV^═O) and (Fe^V^═O) as the main ROS driving the reaction. In particular, the coordination of Fe (III) species in FeN_2_O_2_ to the O atom in the PMS peroxide bond formed a Fe(III)OOSO_3_ complex (Equation 1). The cleavage of the OO– bond within this complex generated Fe^IV^═O (Equation 2), which oxidized PCM through electron transfer, regenerating Fe (III) species in the cycle (Equation 3).

(1) $$\equiv \;\mathrm{Fe}\left(\mathrm{III}\right)+{\mathrm{HSO}}_{4}^{-}+H_{2}O\to {\left[\mathrm{Fe}\left(\mathrm{III}\right)\mathrm{OOS}O_{3}\right]}^{+}+H^{+}$$

(2) $${\left[\mathrm{Fe}\left(\mathrm{III}\right)\mathrm{OOS}O_{3}\right]}^{+}\to \equiv Fe^{\mathrm{IV}}+{\mathrm{SO}}_{4}^{•-}$$

(3) $$\equiv \;Fe^{\mathrm{IV}}=\;O+\mathrm{paracetamol}\to \equiv \mathrm{Fe}\left(\mathrm{III}\right)+\mathrm{products}$$

Direct ETP was also observed in the Fe─N─C/PMS system (Figure 6h). Quenching tests showed that SO_4_^•−^ and ^•^OH contributed minimally (3.5–8.2%) to PCM degradation, with their formation linked to leached Fe ions anchored by pyridinic N. The increase in oxidized species in the spent catalyst (Figure 6i) confirmed the role of pyridinic N in anchoring Fe atoms. The Fe─N─C/PMS system selectively degraded electron‐rich micropollutants, including paracetamol, sulfamethazine and CIP and BPA, while only 20% of chloramphenicol (CP) could be degraded in 20 min (Figure 6j), highlighting the influence of ionization potentials and electronic structure on the reaction. The catalyst also exhibited good reusability for 5 cycles, with heat treatment at 350 °C fully recovering its catalytic ability. Wang et al. synthesized Fe─N─O─C SACs with Fe–N_2_–O_2_ configurations to activate 0.5 mM PMS for sulfamethoxazole (SMX) degradation.^[^ ^104^ ^]^ DFT calculations showed that Fe–N_2_–O_2_ configuration enhanced ^1^O_2_ through the electron delocalization of Fe–O, which lowered the d‐band center of Fe, leading to lower adsorption free energy of the intermediates.^[^ ^144^ ^]^ The resulting activation of PMS facilitated the formation of ^1^O_2_ as the dominant ROS via a stepwise pathway: PMS→OH*→O* →^1^O_2_. This non‐radical mechanism demonstrated a high turnover frequency (TOF) of 1.72 min^−1^ and a removal efficiency of 96.80% within 40 min. Notably, increasing Fe content led to a twofold increase in SMX degradation rates. Comparison studies showed minimal SMX degradation using PMS alone and inactivity with Fe_2_O_3_ nanoparticles, highlighting the catalytic capabilities of the Fe–N_2_–O_2_ configuration. The catalyst maintained >90% SMX removal efficiency after five cycles with Fe leaching below 0.05 mg L^−1^, demonstrating the strength of its coordination structure.

To summarize, multiple atomic Fe coordination sites have been reported on Fe‐based biomass‐derived SACs. Due to different experimental conditions or research methods, similar or different mechanisms have been obtained, e.g., Fe─N_3_─C for ETP,^[^ ^43^ ^]^ Fe─N_4_─C for ETP,^[^ ^56^ ^]^ Fe─N_4_ for ETP and ^1^O_2_,^[^ ^93^ ^]^ Fe–N_2_–O_2_ for ^1^O_2_,^[^ ^104^ ^]^ Fe─N_2_─O_2_ for nonradical high‐valence Fe‐oxo species and ETP.^[^ ^53^ ^]^ Therefore, the single atom of Fe generally promotes nonradical processes, while surface functionalities such as N and O groups, and defects on the carbon support also facilitate PMS activation process.

#### 5.1.2 Biomass‐derived Co‐based SACs for PMS Activation

Biomass‐derived Co‐based SACs often exhibit high activity for PMS activation.^[^ ^119^ ^]^ Liu et al. synthesized Co−N−C SACs with saturated CoN_4_ sites to activate 6 mM PMS through a non‐radical mechanism for degrading Ioxehol (IOH).^[^ ^99^ ^]^ Co–N_4_ sites enhanced PMS adsorption at specific oxygen sites, initiating its decomposition into SO_5_^•−^, and eventually generating ^1^O_2_ as the dominant ROS. DFT calculations indicated that the degradation pathway involves the electrophilic attack of IOH with high Fukui function values, such as iodine atoms and benzene ring. Approximately 0.1 mg L^−1^ of Co^2+^ were leached, well below both Chinese National Standards (GB 25467‐2010) and United States Environmental Protection Agency secondary drinking water standards of 1.0 mg L^−1^, with sole contribution from leached cobalt accounting for only about 10.2% of IOH elimination. The catalyst achieved 98.5% of IOH degradation within 10 min, retaining 88.4% degradation efficiency after five cycles. The Co−N−C SAC/PMS system showed wide applicability for other micropollutants, including pharmaceuticals and organic dyes, personal care products and phenol compounds, specifically, rhodamine B, CIP, BPA, methylene blue, methyl orange, tetracycline hydrochloride (TCH), ENR, SMX and 4‐chlorophenol, all achieving above 75% degradation rate. Xiao et al. used N‐doped walnut shell‐derived biochar to prepare Co−N−C SACs, which activated PMS for sulfadoxin degradation via high‐valent Co═O species.^[^ ^98^ ^]^ The external N doping during synthesis facilitated uniform Co dispersion, preventing nanoparticle formation and enabling enhanced catalytic performance. The system outperformed Co nanoparticles in both ^1^O_2_ generation and selective oxidation of amino groups. Gu et al. applied Co−N−C SACs derived from rice straw enriched with mineral salts for SMX degradation, achieving 99.8% removal within 60 min.^[^ ^97^ ^]^ The ligand‐mediated synthesis anchored Co atoms on defective biochar, forming Co─N_4_ sites coordinated with pyrrolic N. This structure enhanced PMS activation and promoted co‐adsorption of PMS and SMX, enabling efficient ^1^O_2_ generation through ETP. The catalyst demonstrated stability and reusability, achieving a 96.9% removal efficiency after five cycles.

Researchers have also investigated the integration of S to modify the coordination environment of Co centers as a strategy to enhance catalytic performance. For example, Cui et al. synthesized Co─N─S─C SACs with Co−N_3_S_1_ and investigated their performance in activating 2 mM PMS for degrading diethyl phthalate (DEP), 2,4,4 trichlorobiphenyl (PCB28) and BPA.^[^ ^24^ ^]^ The spent coffee grounds served as a dual source of nitrogen and sulfur, enabling the formation of Co^II^─N_3_S_1_ sites through direct coordination with Co ions during pyrolysis. The configuration allowed S to function as an electron transfer mediator between PMS and Co^II^, significantly reducing PMS adsorption energy and enhancing electron transfer. The degradation pathway involved first the binding of HSO_5_^−^ to Co^II^─N_3_S_1_ site, resulting in the generation of SO_4_^•−^ and ^•^OH, while Co^II^ was oxidized to Co^III^. Subsequently, SO_4_^•−^ and carbon/cobalt‐based SACs interactions facilitated the production of ^•^OH and ^1^O_2_. The Co─N─C SAC/PMS system achieved high removal efficiencies: 90% PCB 28 removal within 128 min, 90% DEP degradation in 60 min, and complete BPA degradation in 30 min. For PCB 28, adsorption onto pyrrolic N sites via a donor‐acceptor mechanism was crucial, leading to major degradation by SO_4_^•−^ and ^•^OH, with minimal contribution from ^1^O_2_ (<10%). Only 0.07 mg L^−1^ of Co ions were detected after catalytic reactions, representing a leaching rate of merely 0.62%, significantly lower than conventional Co‐based carbon catalysts.

**Figure 7:**
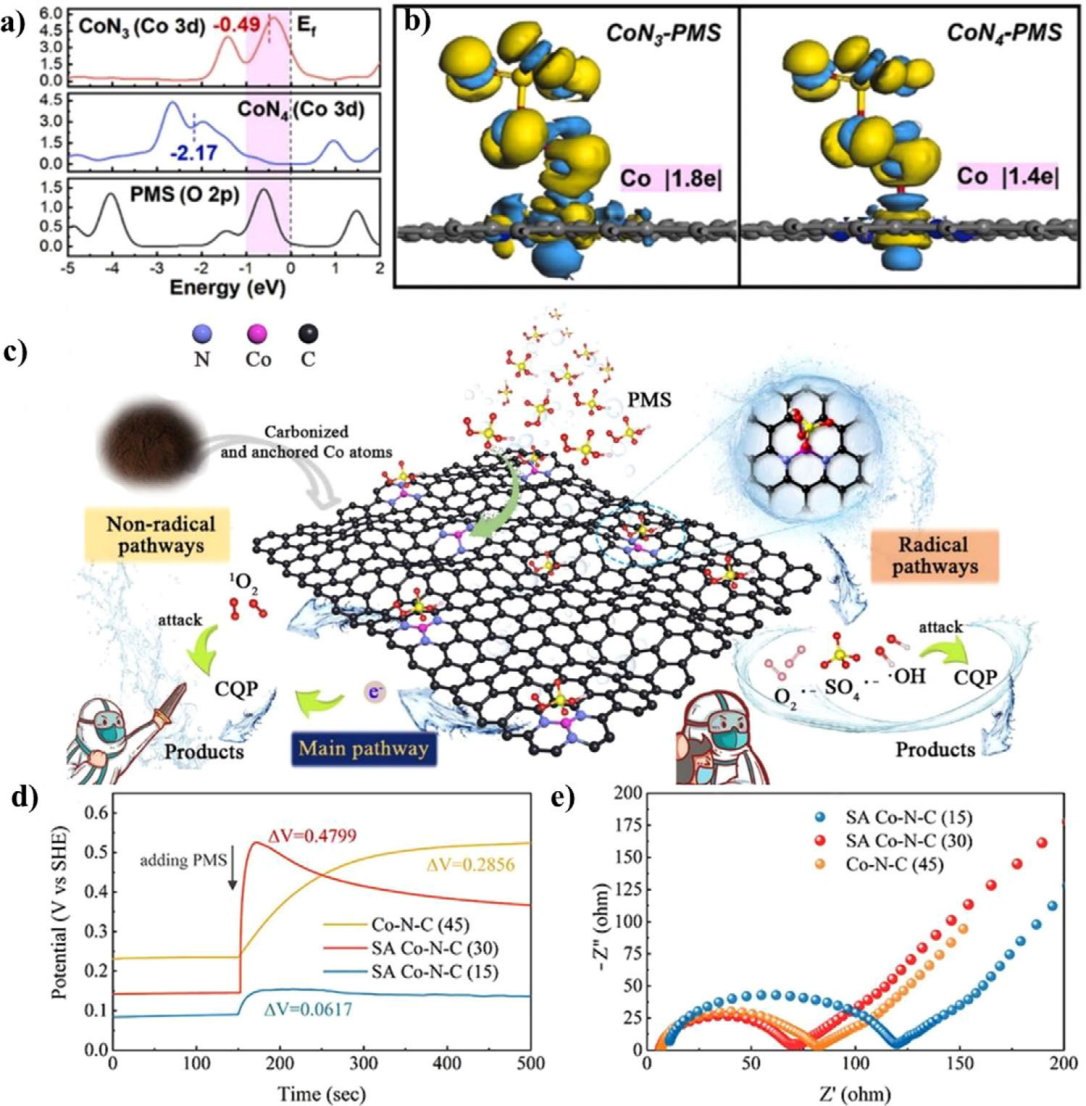
Mechanisms of biomass‐derived Co‐based SACs for the activation of PMS and the degradation of various organic micropollutants. (a) Partial density of states of Co 3d in CoN_3_, Co 3d in CoN_4_ and adsorbed O 2p in PMS. (b) Electron density difference maps of CoN_3_‐PMS (left) and CoN_4_‐PMS (right). Yellow colors and light blue colors represent the electron accumulation and electron depletion, respectively. The exposed black and dark blue balls represent C and N atoms, while the Co atoms are wrapped in electron clouds. Reproduced with permission.^[^ ^55^ ^]^ Copyright 2023, Elsevier. (c) The proposed mechanism for Co−N−C SAC/PMS system. (d) Open‐circuit potentials and (e) EIS Nyquist plots of Co−N−C SAC/PMS complexes with different Co loadings. Reproduced with permission.^[^ ^69^ ^]^ Copyright 2022, Elsevier.

CoN_3_ coordination has also been identified as an important structural feature for enhanced catalytic performance. For instance, in one study, Co−N−C SACs with CoN_3_ sites acted as major active centers for activating 0.8 mM PMS, facilitating complete degradation of Rhodamine B within 5 min.^[^ ^55^ ^]^ The Co−N−C SACs were derived from chitosan, a N‐rich biopolymer whose abundant amine groups enabled effective chelation with Co^2+^ ions and supported the formation of unsaturated CoN_3_ sites during pyrolysis. The higher D‐band center of CoN_3_ (−0.49 eV) compared to that of CoN_4_ (−2.17 eV), showed stronger adsorption and interaction with PMS (**Figure** **7**a), as supported by DFT calculations. This electronic property facilitated enhanced electron flow and enabled the generation of high‐valent Co (IV) species, which were pivotal in PMS activation. Further, the unsaturated CoN_3_ sites, with a higher spin state, efficiently activated PMS through enhanced electron flow compared to CoN_4_ sites (Figure 7b), leading to the formation of Co (IV) instead of only free radicals (^•^OH and SO_4_^•−^) in the CoN_4_ system. Peng et al. demonstrated that CoN_3_ unsaturated sites in Co−N−C SACs serve as the primary active centers for PMS activation, achieving 97.5% CQP degradation within 30 min using 1 mM PMS.^[^ ^69^ ^]^ The Co−N−C SACs, derived from lignin, benefited from Zn‐assisted pyrolysis, which helped disperse Co atoms and enhance porosity. The resulting curly sheet carbon morphology with SSA (305.33 g m^2^ g^−1^) allowed effective diffusion of PMS and CQP to the active CoN_3_ sites. These structural characteristics supported the formation of surface‐bound active complexes, which enhanced direct electron transfer from CQP to PMS (Figure 7c). The optimized Co loading and coordination environment enabled selective degradation with ETP as the dominant pathway, as confirmed by Open‐circuit potential measurements (Figure 7d). Electrochemical impedance spectroscopy (EIS) analysis revealed that Co−N−C SACs with higher Co content exhibited smaller Nyquist plots, indicating lower charge transfer resistance (Figure 7e). This improvement was attributed to the higher pyridine N content, which strengthened the interaction between PMS and the catalyst. Such optimization enhanced electron transfer efficiency, boosting the degradation rate of CQP. Qi and co‐workers fabricated a lignin‐based Co─N─C SACs that facilitated 0.5 mM PMS activation for NPX degradation through ETP.^[^ ^25^ ^]^ The use of lignin, rich in phenolic and hydroxyl groups, allowed for stable metal‐biomass complexation, while Zn salt evaporation during pyrolysis promoted atomic Co dispersion and mesopore formation. These synthesis features led to the formation of unsaturated CoN_3_(II) sites that, due to their electron‐rich pyridinic coordination, enabled direct interaction with PMS. As a result, complete NPX degradation was achieved within 90 min, with a high TOF of 4.82 min^−1^. ROS such as ^1^O_2_, ^•^OH and SO_4_^•−^ contributed minimally to the process, showing performance reliance on the electronic properties and accessibility of the CoN_3_ active sites shaped by the biomass‐derived structure. The catalyst showed universal application in degrading a range of micropollutants, including BPA, CIP, metronidazole (MNZ), NPX and PCM. While NPX, PCM, CIP, and BPA were degraded completely (k of 0.18‐0.35 min^−1^), only 40–60% MNZ and CP were removed within 90 min. However, even with optimized single‐atom structures, approximately 5% Co leaching was observed after multiple cycles.

In conclusion, various studies have documented the different Co coordination sites for Co‐based biomass‐derived SACs. However, differing experimental approaches have led to variations in the mechanisms obtained, e.g., Co−N_4_ for high valent Co═O,^[^ ^98^ ^] 1^O_2_,^[^ ^99^ ^]^ or ^•^OH and SO_4_^•−^;^[^ ^55^ ^]^ Co−N_3_ for ETP,^[^ ^25^, ^69^ ^]^ or high valent Co(IV);^[^ ^55^ ^]^ Co−N_3_S_1_ for ^•^OH, SO_4_^•−^,^[^ ^24^ ^]^ and octahedral Co and pyrrolic N for ^1^O_2_ and ETP.^[^ ^97^ ^]^ It can be inferred that unsaturated Co−N_3_ coordination tends to participate in electrophilic interactions and induces non‐radical processes, while Co−N_4_ can catalyze both radical and non‐radical pathways.

#### 5.1.3 Biomass‐derived Cu‐based SACs for PMS Activation

**Figure 8:**
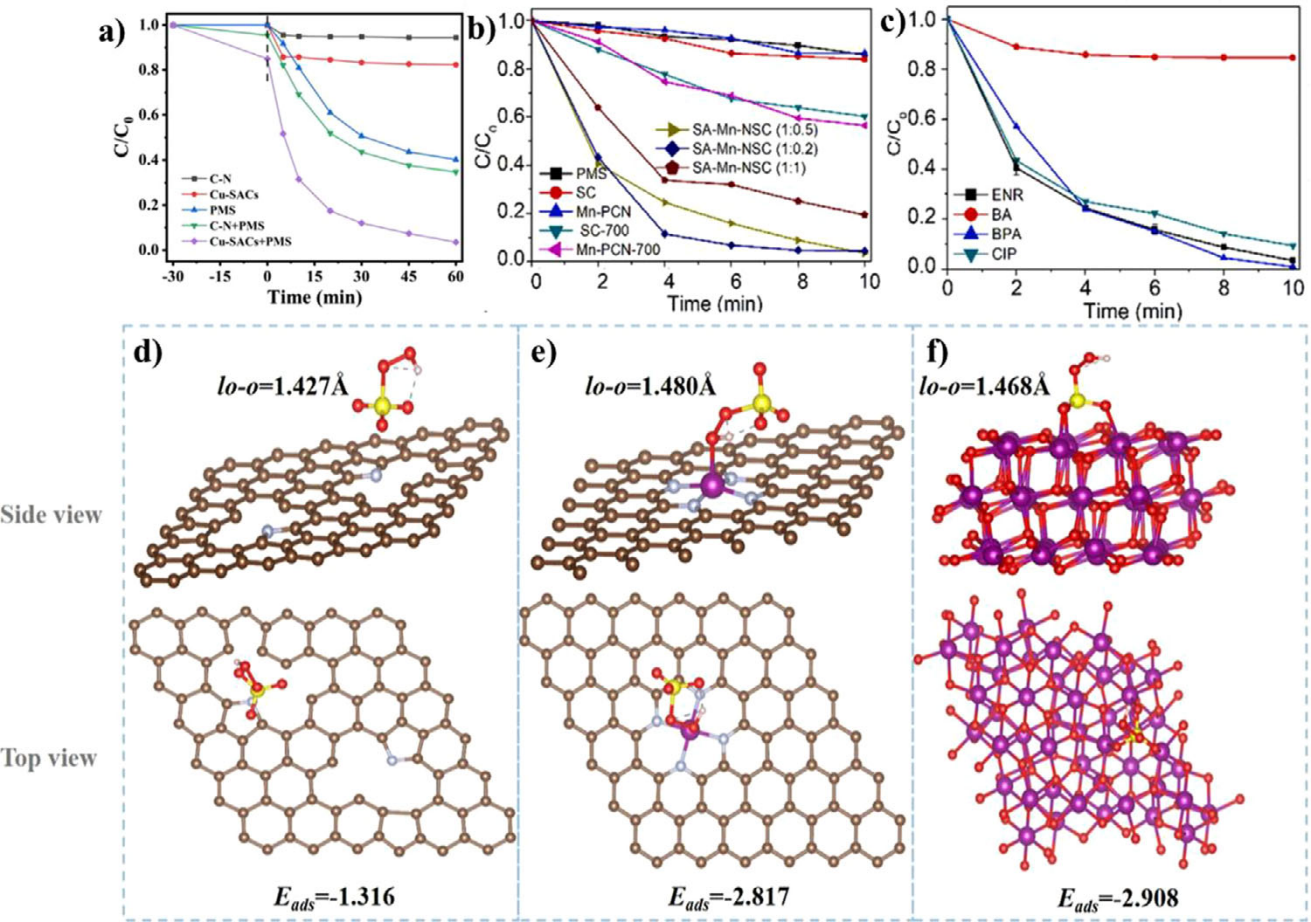
Biomass‐derived Cu‐ and Mn‐based SACs for the activation of PMS and the degradation of various organic micropollutants. (a) Adsorption and degradation curves of different materials. Reproduced with permission.^[^ ^58^ ^]^ Copyright 2022, Elsevier. (b) ENR degradation by different Mn─N─C SAC/PMS systems and (c) degradation curves of various micropollutants. Reproduced with permission.^[^ ^51^ ^]^ Copyright 2023, Elsevier. DFT calculations of (d) N‐doped graphene, (e) single atom Mn─N_4_ sites, and (f) Mn_2_O_3_(222). Reproduced with permission.^[^ ^63^ ^]^ Copyright 2023, American Chemical Society.

Cu‐based SACs with different active sites have shown ability to activate PMS for degrading micropollutants. Pan et al. synthesized Cu─N─C SACs from a waste adsorbent and reported single‐atom Cu in Cu–N_4_ coordination as the active site in PMS activation for degrading BPA in high‐salinity wastewater.^[^ ^118^ ^]^ The catalyst demonstrated 100% removal of BPA within 30 min and ETP was determined to be the dominant pathway. The direct anchoring of single‐atom Cu on the carbon support via dative bonds enhanced conductivity of Cu─N─C SACs and PMS adsorption capacity. Among the four Cu─N─C SACs, the catalyst performance increased with higher atomic Cu amount. Cu─N─C SACs maintained >97% removal efficiency over five cycles with Cu leaching below 0.5%, demonstrating the stability of Cu─N_4_ coordination in biochar. With similar Cu─N_4_ sites, Liu et al. showed that Cu─N─C SACs synthesized from α‐cellulose enabled 96.47% OTC degradation in 60 min (**Figure** **8**a) using 0.05 mM PMS.^[^ ^58^ ^]^ Phenanthroline acted as both N source and chelating ligand to form Cu─N_4_ sites. Detection of ^1^O_2_ and ^•^OH highlighted the dual‐pathway mechanism, with catalyst performance closely tied to Cu atom loading and the structural integration of Cu─N coordination within the biomass‐derived matrix. After three cycles, degradation efficiency dropped to 63.98%, primarily attributed to the leaching of unstable Cu active sites from the catalyst surface. This highlights the need for strategies to enhance Cu^2+^ conversion and stabilize active sites for improved cyclability.

#### 5.1.4 Biomass‐derived Mn‐Based SACs for PMS Activation

Mn‐based SACs are one of the most promising catalysts for Fenton‐like reactions due to their various valence states (Mn^2^⁺, Mn^3^⁺, Mn⁴⁺, and Mn⁷⁺), which allow Mn atoms to easily switch between different redox states, making them highly effective in catalyzing Fenton‐like reactions. Zhou et al. developed Mn─N─S─C SACs with Mn─N_4_ coordination for degrading ENR. The use of protein‐rich spirulina as a precursor, combined with dicyandiamide, enabled strong Mn─N coordination, forming atomically dispersed Mn─N_4_ sites.^[^ ^51^ ^]^ This facilitated 1 mM PMS activation and selective ^1^O_2_ and O_2_^•−^ generation, achieving over 93% ENR removal in real wastewater matrices. A direct correlation was observed between Mn─N─S─C SACs and Mn‐doped polymeric carbon nitride (MN‐PCN) (Figure 8b), confirming single‐atom Mn as catalytic centers for PMS activation. The Mn─N─S─C SACs/PMS demonstrated a high specificity for micropollutants with phenolic hydroxyl groups (Figure 8c). The Mn─N─S─C SAC/PMS system showed promising stability with Mn leaching below 0.07 mg L^‒1^ during operation. This relatively low leaching rate suggests effective coordination of Mn atoms within the carbon framework, presenting a potential approach for developing stable SACs for water treatment. Yang et al. employed *P‐americana*, a metal‐accumulating biomass, to develop Mn─N─C SACs with Mn‐pyrrolic N_4_ coordination.^[^ ^63^ ^]^ Stepwise pyrolysis at increasing temperatures enhanced Mn stabilization by promoting graphitization of the carbon matrix. These structural changes enabled high CQP adsorption near reactive Mn‐pyrrolic N_4_ centers, reducing ROS migration distances and enhancing degradation. Mn─N─C SACs activated 2 mM PMS to remove >90% of CQP within 30 min. The combination of radical (^•^OH, SO_4_^•−^) and non‐radical (^1^O_2_) pathways was enabled by the atomic dispersion of Mn and coordination with N dopants from biomass and urea, linking thermal control and precursor design to catalytic performance. DFT calculations revealed PMS activation mechanisms across different catalytic sites (Figure 8d–f). Upon PMS addition, the O−O bond length increased from 1.322 Å, and the single atom Mn─N_4_ sites exhibited the most significant bond elongation to 1.480 Å compared to pristine N‐doped graphene (1.427 Å) and Mn_2_O_3_ (222) (1.468 Å) surfaces, demonstrating that the Mn─N_4_ sites facilitated PMS activation. Despite maintaining Mn leaching below 0.02 mg L^‒1^ across multiple cycles, the Mn─N─C SAC/PMS system showed decreased efficiency for CQP degradation, dropping to 58.86% and 61.15% in the second and third cycles, respectively. This performance decline was attributed not primarily to metal leaching but to catalyst recovery losses and surface fouling by adsorbed reaction intermediates, highlighting the varied nature of catalyst deactivation in practical applications.

In conclusion, studies on PMS activation have demonstrated that the coordination of transition metals, such as Fe, Co, Mn, and Cu, with heteroatoms (N, S, O), plays a crucial role in PMS activation. Biomass‐derived SACs combine the unique features of carbon supports and monoatomic catalysts, where different types of metal coordination and transition metal species can lead to distinct reaction pathways. These pathways are further influenced by the properties of the carbon support, such as SSA, porous structure, and heteroatoms. Together, these properties create synergistic effects that significantly enhance the catalytic process. Due to varying research methods, metallic species, coordination structures, and support characteristics, multiple mechanisms have been observed for PMS activation. It is recommended that systematic studies be undertaken to further elucidate the interconnected relationships and synergistic effects of coordination structures, metal atom centers, carbon support properties, structural vacancies, defective sites, and heteroatoms in single‐atom catalysis‐driven PMS activation. Overall, the metal atom centers and their associated coordination sites are key to the design and optimization of SACs for Fenton‐like reactions, directly influencing the efficiency of the degradation process.

### 5.2 PDS Activation

Compared to the asymmetric structure in PMS, PDS possesses a symmetric structure (SO_3_─O─O─SO_3_),^[^ ^128^, ^145^, ^146^, ^147^, ^148^, ^149^ ^]^ making it more stable and requiring higher energy for activation. Both PMS and PDS are widely used as oxidants for water remediation studies involving SACs.

#### 5.2.1 Biomass‐Derived Fe‐Based SACs for PDS Activation

Fe‐based SACs can activate PDS to generate ROS, which are essential in degrading micropollutants using specific active sites. For a Fe─N─C SAC/PDS system, Wang et al. investigated Fe─N─C SACs derived from *Enteromorpha* as catalysts for PDS activation.^[^ ^109^ ^]^ The Fe‐pyridinic N_4_ moiety in the SACs served as the primary coordination center, enhancing PDS adsorption and facilitating ETP‐based degradation of 4,4‐sulfonyl diphenol (BPS). This system achieved complete BPS degradation within 40 min, with no degradation observed from direct PDS. Electrochemical analysis and theoretical calculations confirmed that Fe‐pyridinic N_4_ sites were pivotal as electron mediators, forming Fe─N─C SAC/PDS* complexes that acted as oxidizing intermediates. In contrast, pyrrolic FeN_4_ sites contributed minimally to PDS activation, highlighting the specificity of coordination effects. Probe tests, ESR analysis, and quenching experiments ruled out SO_4_^•−^ or ^•^OH as the dominant ROS in this system. Rather, the degradation pathway was primarily non‐radical, driven by the ETP mechanism facilitated by the Fe‐pyridinic N_4_ coordination. The Fe─N─C SAC/PDS system demonstrated substrate specificity in degradation rates, with electron‐donating contaminants showing faster degradation than electron‐withdrawing contaminants. The catalyst showed extended applicability, achieving above 90% in both surface water and tap water. Furthermore, it exhibited sustained performance, maintaining 70% BPS degradation after 20 h of operation.

#### 5.2.2 Biomass‐Derived Co‐Based SACs for PDS Activation

Both radical and non‐radical pathways can be induced in Co─N─C SAC/PDS systems with unsaturated Co─Nx sites. Qian et al. employed wastepaper functionalized with ‐COOH groups and doped with Co and PEI‐derived nitrogen to fabricate Co─N─C SACs with Co─N_3_ coordination sites.^[^ ^122^ ^]^ The Co─N_3_ coordination facilitated the cleavage of the PDS O─O bond, generating SO_4_^•−^ as the primary ROS (Equations (4), (5), (6)). Simultaneously, ^•^OH was produced through redox reactions (Equations 6 and 8), with the Co^2+^/Co^3+^ redox cycle sustaining the catalytic process.

(4) $$\equiv \;Co^{2+}+S_{2}O_{8}^{2-}\to \equiv Co^{3+}+{\mathrm{SO}}_{4}^{•-}+{\mathrm{SO}}_{4}^{2-}$$

(5) $$S_{2}O_{8}^{2-}+H_{2}O\to {\mathrm{HSO}}_{5}^{-}+{\mathrm{HSO}}_{4}^{-}$$

(6) $$\equiv \;Co^{2+}+{\mathrm{HSO}}_{5}^{-}\to \equiv Co^{3+}+{\mathrm{SO}}_{4}^{•-}+OH^{-}$$

(7) $$\equiv \;Co^{3+}+{\mathrm{HSO}}_{5}^{-}\to \equiv Co^{2+}+{\mathrm{SO}}_{5}^{•-}+H^{+}$$

(8) $${\mathrm{SO}}_{4}^{•-}+H_{2}O\to {\mathrm{HSO}}_{4}^{-}+•\mathrm{OH}/{\mathrm{SO}}_{4}^{2-}+•\mathrm{OH}+H^{+}$$

The Co─N_3_ sites also served as electron shuttles in ETP, with valence electrons from unsaturated Co─N_3_ interacting with PDS to facilitate degradation. Within 1 min, the Co─N─C SAC/PDS system achieved 93% SIZ removal efficiency, highlighting the effectiveness of unsaturated Co─N_3_ coordination in promoting PDS activation and ROS generation. Further, the Co─N─C SAC/PDS system exhibited stability with Co leaching below 0.05 mgL^−1^ across a wide pH range (3‐11), demonstrating its robustness in different water conditions.

**Figure 9:**
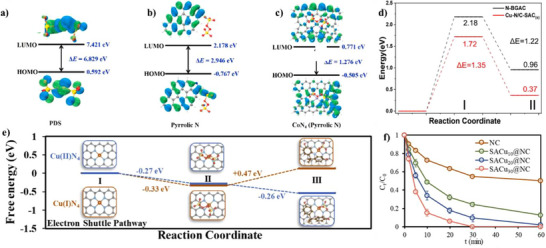
Mechanisms of biomass‐derived SACs for the activation of PDS and degradation of various organic micropollutants. HOMO and LUMO of (a) PDS, (b) pyrrolic N/PDS and (c) CoN_4_ (pyrrolic N)/PDS for Co─N─C SACs. Reproduced with permission.^[^ ^119^ ^]^ Copyright 2023, Elsevier. PDS adsorption and activation processes of N‐doped biochar and Cu─N─C SACs based on DFT calculation: (d) Potential energy profiles. Reproduced with permission.^[^ ^84^ ^]^ Copyright 2024, Elsevier (e) Reaction pathways of PDS activation and BPA oxidation at Cu sites with different valences via electron shuttle process (orange and blue routes represented reaction on Cu(I)N_4_ and Cu(II)N_4_ sites, and the green box represents the common structure) and (f) The degradation curves of BPA in different Cu─N─C SAC/PMS systems. Reproduced with permission.^[^ ^125^ ^]^ Copyright 2024, Elsevier.

Zhang et al. demonstrated that CoN_4_ (pyrrolic N) sites in the synthesized Co─N─C SACs from straw biochar facilitated PDS activation for TCH degradation.^[^ ^119^ ^]^ These sites promoted ETP, allowing efficient electron transfer from TCH to PDS. The involvement of the CoN_4_ (pyrrolic N) sites was pivotal in forming Co─N─C SAC/PDS complexes, which facilitated the binding and activation of PDS. This interaction was supported by molecular orbital studies, revealing that the LUMO energy of the CoN_4_ (pyrrolic N)/PDS complex (0.771 eV) was significantly lower than PDS alone (7.421 eV) or pyrrolic N/PDS (2.178 eV) (**Figure** **9**a‐c). This lower LUMO value enhanced the oxidative ability of the complex, making it more effective at accepting electrons from TCH. In addition to ETP, the carbonyl group in the Co─N─C SAC structure played a role in generating ^1^O_2_ as the primary ROS for antibiotic degradation. This dual mechanism highlights the versatility of the CoN_4_ (pyrrolic N) coordination environment. This system achieved 100% degradation of TCH in 6 min, demonstrating efficiency in micropollutant degradation. Co─N─C SACs exhibited superior conductivity to other catalysts, facilitating electron transfer for PDS activation.

#### 5.2.3 Biomass‐Derived Cu‐Based SACs for PDS Activation

Cu‐based SACs have been explored to activate PDS and enable the generation of ROS for degrading various organic micropollutants. For example, Cu─N─C SAC/PDS system exhibited a TC removal efficiency of 87% within 90 min.^[^ ^84^ ^]^ These Cu─N─C SACs were synthesized using blue‐green algal biomass, which provided nitrogen‐containing functional groups, facilitating Cu anchoring during molten salt‐assisted pyrolysis. The degradation was enhanced by the adsorption of PDS onto Cu─N─C sites, where single‐atom Cu facilitated electron transfer to activate PDS. This activation generated ^1^O_2_ and O_2_^•−^ (Equations 9 and 10) as the dominant ROS with ^•^OH and SO_4_^•−^ playing minor roles. DFT calculations revealed lower PDS adsorption energy on Cu─N─C SACs (1.78 eV) compared to N‐doped biochar (2.18 eV, Figure 9d). Further, the well‐dispersed Cu atoms primarily in their divalent state, were coordinated with two N and two C atoms, forming a stable CuN_2_C_2_ structure. The catalyst also demonstrated recyclability after 4 cycles, achieving TC removal efficiency of 71.8%.

(9) $$\begin{array}{c} Cu-N-C\;SAC+2H_{2}O+2S_{2\;}O_{8}^{2-}\to Cu-N-C\;SAC\\ +\;4SO_{4}^{2-}{+}^{1}O_{2}+4H^{+} \end{array}$$

(10) $$Cu-N-C\;SAC\;\left(e^{-}\right)+O_{2}\to Cu-N-C\;SAC+O_{2}^{•-}$$

Pan et al. investigated the performance of Cu─N─C SACs with Cu (I) and Cu (II) species in CuN_4_ coordination for PDS activation for efficient BPA degradation, driven by Cu valence state changes.^[^ ^125^ ^]^ Cu (I) reacted with adsorbed PDS to generate Cu (III), and SO_4_^•−^ at CuN_4_ sites, while Cu (II) could form a complex with PDS to oxidize BPA through ETP directly (Figure 9e). A relatively higher Cu content (2.8‐3.5%) in Cu─N─C SACs enhanced BPA degradation rate (Figure 9f), with a strong positive correlation (R^2^ = 0.94). Cu─N─C SACs showed promising practical application potential with Cu leaching initially at 80 *µ*g L^−1^ during continuous‐flow experiments.

To sum up, the choice of metals (Fe, Co, Cu) in SACs for Fenton‐like catalysis significantly influences the PDS activation mechanisms and efficiency of pollutant degradation by radical or nonradical pathways. Factors such as the metal's redox potential, coordination environment, interaction with the support material, and heteroatoms play pivotal roles in determining the catalyst's performance. These findings demonstrate the significance of tailoring the coordination environment of transition metal‐based SACs to effectively activate PDS for Fenton‐like catalysis applications.

### 5.3 Biomass‐Derived SACs for H_2_O_2_ Activation

H_2_O_2_ is a potent oxidant, and its activation usually results in the generation of ROS such as ^•^OH. Hou et al. applied Fe─N─C SACs to activate 0.3 mM H_2_O_2_ for degrading SMX.^[^ ^65^ ^]^ Fe─N─C SACs were synthesized from Chlorella vulgaris, a N‐rich biomass, which provided abundant N functionalities that promoted the formation of Fe─N coordination sites and high SSA. According to the DFT calculations, Fe/Fe compounds markedly enhanced the activity of atomic Fe─Nx by facilitating the transfer of electrons between Fe─Nx and the oxidants (H_2_O_2_ and O_2_). SMX was completely degraded within 30 min in the Fe─N─C SAC/H_2_O_2_ system, while only 6.52% was removed in the biochar/H_2_O_2_ system. The ROS involved in the degradation of SMX by Fe─N─C SAC/H_2_O_2_ were ^•^OH, ^1^O_2_ and O_2_^•−^. The Fe─N─C SAC/H_2_O_2_ system demonstrated universality in the efficient degradation of carbamazepine, sulfadiazine, phenol, CIP and NPX.

**Scheme 2:**
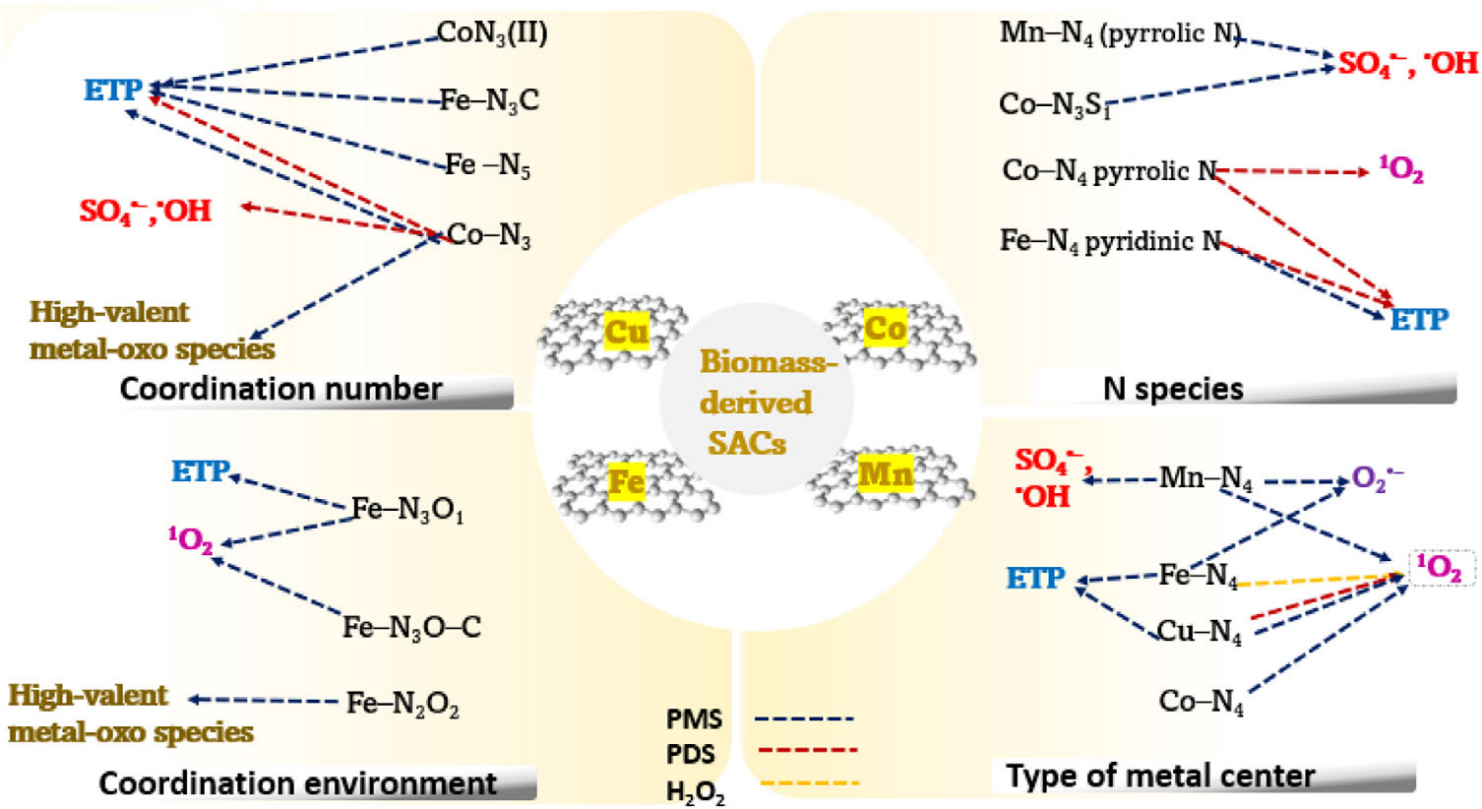
Effect of different active sites with different coordination number, coordination environment, N species and type of metal center on degradation pathways in Fenton‐like reactions for different biomass‐derived SACs.

Overall, the specific identification of the degradation pathway or mechanism in Fenton‐like reactions can be challenging due to the generation of multiple ROS, competing reaction pathways, changes in metal oxidation states, heterogeneous catalytic systems, and the water matrix. However, the design of active sites is essential for optimal catalytic efficiency and is collectively centered on tailoring the specific N species, spin state and coordination environment of the metal. **Scheme** **2** provides an overview of the various factors contributing to the generation of different ROS in Fenton‐like reactions for different biomass‐derived SACs.

## 6 Conclusions and Future Perspectives

The development of biomass‐derived SACs for Fenton‐like reactions represents a significant advancement in sustainable environmental remediation technologies. These catalysts combine the advantages of atomic‐level catalytic efficiency with the renewable nature of biomass precursors, offering an eco‐friendly alternative to conventional catalytic systems. Through various synthesis approaches, including direct pyrolysis, template‐assisted methods, and chemical activation, researchers have successfully developed SACs with well‐defined metal coordination environments (M−N_4_, M−N_3_, etc.) and tailored textural properties. These structural features are crucial for their exceptional performance in activating oxidants like PMS, PDS, and H_2_O_2_ for organic micropollutant degradation.

The application of biomass‐derived SACs in Fenton‐like reactions has revealed important structure‐activity relationships. The atomic dispersion of metal centers and their specific coordination environments significantly influence the reaction pathways, enabling both radical and non‐radical mechanisms. These insights have guided the rational design of more efficient catalysts, though a more comprehensive understanding of the underlying mechanisms is still needed, particularly for H_2_O_2_ activation systems, which remain less explored compared to PMS/PDS systems.

Despite these advances, several challenges must be addressed to realize the full potential of biomass‐derived SACs. The synthesis processes need further optimization to achieve higher metal loadings while maintaining atomic dispersion, and to improve batch‐to‐batch reproducibility, given the heterogeneous nature of biomass feedstocks. Stability issues under operational conditions should be investigated. The development of standardized characterization methods will be essential for better comparing and optimizing different catalyst systems.

Looking forward, the field should focus on bridging the gap between laboratory research and practical implementation. This includes developing scalable and cost‐effective synthesis methods, designing continuous production processes, and conducting comprehensive life‐cycle assessments. Future research should adopt a multidisciplinary approach combining advanced materials characterization, computational modelling, and engineering principles. Particular attention should be paid to system integration for real‐world water treatment applications and the development of hybrid processes that combine SACs with other advanced oxidation technologies. By addressing these challenges, biomass‐derived SACs can transition from promising laboratory discoveries to practical solutions for sustainable water purification and environmental remediation.

## Conflict of Interest

The authors declare no conflict of interest.
